# Blood Pressure Control Among Black and White Adults Following a Quality Improvement Program in a Large Integrated Health System

**DOI:** 10.1001/jamanetworkopen.2022.49930

**Published:** 2023-01-06

**Authors:** Teresa N. Harrison, Hui Zhou, Rong Wei, Jeffrey Brettler, Paul Muntner, Jaejin An, Angeline L. Ong-Su, Kristi Reynolds

**Affiliations:** 1 Department of Research & Evaluation, Kaiser Permanente Southern California, Pasadena; 2 Department of Health Systems Science, Kaiser Permanente Bernard J. Tyson School of Medicine, Pasadena, California; 3 Kaiser Permanente West Los Angeles Medical Center, Los Angeles, California; 4 Department of Epidemiology, University of Alabama at Birmingham; 5 Kaiser Permanente Panorama City Medical Center, Panorama City, California

## Abstract

**Importance:**

A higher percentage of non-Hispanic Black (hereinafter, Black) adults vs non-Hispanic White (hereinafter, White) adults with hypertension have uncontrolled blood pressure (BP) contributing to racial and ethnic disparities in cardiovascular disease. In 2010, Kaiser Permanente Southern California began implementing quality improvement (QI) strategies aimed at reducing this disparity.

**Objective:**

To examine the change in BP control between Black and White patients before and after the implementation of a QI program.

**Design, Setting, and Participants:**

A QI quasi-experimental, difference-in-difference analysis was conducted of Kaiser Permanente Southern California patients 18 years or older included in the population care management hypertension registry. The study was conducted from December 31, 2008, to December 31, 2019. Data analysis was performed from November 20, 2020, to November 7, 2022.

**Interventions:**

Quality improvement program implementation began in 2010.

**Main Outcomes and Measures:**

Blood pressure control (systolic BP <140 mm Hg and diastolic BP <90 mm Hg) was assessed using the last outpatient BP measurement in each calendar year. Changes in BP control between Black and White patients from before (2008-2009) to after (2016-2019) implementation of the QI program were examined using a difference-in-difference analysis. Blood pressure control disparities from 2008 through 2019 by age, sex, race and ethnicity, and factors associated with BP control were examined.

**Results:**

The number of patients with hypertension increased from 624 094 in 2008 (mean [SD] age, 61.8 [13.5] years; 330 551 [53.0%] female patients; 89 407 [14.3%] Black and 284 116 [45.5%] White patients) to 855 257 in 2019 (mean [SD] age, 64.5 [13.6] years; 444 422 [52.0%] female patients; 107 054 [12.5%] Black and 331 932 [38.8%] White patients). Blood pressure control increased an absolute 4.6% (95% CI, 4.3%-4.8%) among Black patients and 2.1% (95% CI, 2.0%-2.2%) among White patients from before to after the QI program implementation (difference-in-difference: 2.5%; 95% CI, 2.2%-2.8%). The largest reduction in BP control disparity between Black and White female patients was for those aged 50 to 64 years (difference-in-difference: 3.8%; 95% CI, 3.2%-4.4%) and for those aged 18 to 49 years between Black and White male patients (difference-in-difference: 4.2%; 95% CI, 3.0%-5.5%). The proportion of BP control among Black male patients aged 18 to 49 years was the lowest throughout 2008-2019 compared with male and female patients in other age and racial and ethnic groups. In 2019, uncontrolled BP was more common among Black vs White patients (prevalence ratio: 1.13; 95% CI, 1.12-1.14).

**Conclusions and Relevance:**

This QI program noted that disparities in BP control between Black and White patients were decreased but not eliminated following implementation of QI strategies aimed at reducing disparities in BP control. These findings suggest that more focused interventions may be needed to increase BP control among Black individuals.

## Key Points

**Question:**

Was the blood pressure (BP) control disparity reduced between Black and White patients following implementation of a quality improvement program?

**Findings:**

This quality improvement study of adults with hypertension from 2008 (n = 624 094) to 2019 (n = 855 257) noted that the disparity in BP control between Black and White patients was reduced from before to after the quality improvement program, although not eliminated. Black male patients aged 18 to 49 years had the largest BP control disparity and the lowest proportion of BP control compared with Black and White male and female patients in other age groups.

**Meaning:**

The findings of this study suggest that more focused quality improvement strategies are needed to increase BP control among younger Black patients.

## Teaser

This quality improvement study examines changes in blood pressure control between Black and White patients after use of a quality improvement program.

## Introduction

Hypertension is the leading preventable risk factor for cardiovascular disease (CVD) in the US, and randomized clinical trials have shown that lowering blood pressure (BP) with antihypertensive medication reduces the risk of CVD.^1,2^ There is substantial evidence demonstrating that non-Hispanic Black (hereinafter, Black) adults with hypertension in the US are less likely to have controlled BP compared with non-Hispanic White (hereinafter, White) adults with hypertension.^3,4,5,6^

Since 2004, Kaiser Permanente Southern California (KPSC), a large, integrated health care delivery system, has had a multifaceted quality improvement (QI) hypertension program including a population care management registry to improve identification and treatment of patients with hypertension.^7^ Although BP control for the overall KPSC population with hypertension increased following implementation of the QI program, racial and ethnic disparities were identified in 2009, with Black patients being less likely than White patients to have BP control.^8^ In 2010, KPSC began using Healthcare Effectiveness Data and Information Set performance measures, stratified by race and ethnicity, as part of a national Kaiser Permanente program to address the gap in BP control between Black and White patients.^8^ Medical centers within the KPSC region implemented various approaches from 2010 to 2015 to address BP control disparities and strengthen hypertension care management. Strategies included clinician and staff education programs around building trust and integrating culturally tailored communication tools, including use of the Acknowledge, Introduce, Duration, Explanation communication model,^9^ African American story telling videos,^10^ large group sessions, patient education emphasizing a low-sodium Dietary Approaches to Stop Hypertension diet, smoking cessation, exercise, weight reduction, medication adherence, and other changes listed in eTable 1 in the Supplement.

In the present QI study, we examined the change in BP control among Black and White patients from before (2008-2009) to after (2016-2019) the implementation of the hypertension QI initiative using a difference-in-difference analysis. We also report BP control disparities from 2008 through 2019 by age, sex, and race and ethnicity and examined factors associated with BP control.

## Methods

The study protocol was reviewed and approved by the KPSC Institutional Review Board, and a waiver of written informed consent was obtained due to the retrospective nature of the study. This study followed the Strengthening the Reporting of Observational Studies in Epidemiology (STROBE) reporting guideline for cohort studies.

### Setting

The KPSC health care system currently provides care to 4.8 million members at 15 medical centers and more than 230 medical offices. The membership is diverse and representative of the general population of Southern California.^11^ All aspects of medical care are captured through clinical and administrative databases and an electronic health record.

### Study Design

We included patients who were identified as having hypertension by the KPSC population care management hypertension registry between December 31, 2008, and December 31, 2019. Only patients 18 years or older who meet 1 or more of the following criteria are included in the hypertension registry: (1) 2 outpatient visits within 1 year with a diagnosis code for or related to hypertension (*International Classification of Diseases, Ninth Revision*, or *International Statistical Classification of Diseases and Related Health Problems, Tenth Revision* [eTable 2 in the Supplement]); (2) 1 outpatient visit with a diagnosis code for hypertension and 1 hospital discharge with a diagnosis code for hypertension within 1 year of each other; (3) 1 antihypertensive medication (α-blocker, α-2 receptor agonist, angiotensin-converting enzyme inhibitor, angiotensin II receptor blocker, β-blocker, calcium channel blocker, or thiazide, potassium-sparing, or loop diuretic) dispensed in the previous 6 months and 1 outpatient visit with a diagnosis code for hypertension within 1 year prior to the dispense date; or (4) 1 outpatient visit with a diagnosis code for hypertension and a history of heart failure, diabetes, coronary artery disease, chronic kidney disease (CKD), or cerebrovascular disease. Patient demographic characteristics, including age, sex, and race and ethnicity, were collected from the electronic health record. Race and ethnicity were self-reported at clinic visits and categorized into mutually exclusive groups, including Hispanic regardless of race and the following non-Hispanic groups: Asian or Pacific Islander, Black, White, and other (defined as Native American or Alaska Native and multiple or other races and ethnicities). Data for Asian or Pacific Islander, Hispanic and other racial and ethnic groups are reported in eTables 3 though 10 and eFigures 1 through 3 in the Supplement. Comorbidities, including CVD, CKD, heart failure, and diabetes, were based on a combination of diagnosis codes and laboratory values identified from any point prior to a patient’s entry into the hypertension registry.

Blood pressure control was based on the last available BP reading in each calendar year from 2008 through 2019. Blood pressure control was defined as systolic BP less than 140 mm Hg and diastolic BP less than 90 mm Hg.^12^ If a patient did not have any BP measurements during a calendar year (4.9% of Black patients and 4.2% of White patients in 2008-2009 and 5.8% of Black patients and 5.6% of White patients in 2016-2019), the patient was considered to have uncontrolled BP during that year. Antihypertensive medication use was defined as a patient filling 1 of the 7 medication classes noted above alone or in combination in each year in which they were included in the hypertension registry

### Statistical Analysis

Data analysis was performed from November 20, 2020, to November 7, 2022. The demographic and clinical characteristics of patients with hypertension were described by calendar year from 2008 to 2019, and the characteristics were described separately by race and ethnicity for 2008 and 2019. The age-adjusted estimated proportion of adults with BP control was calculated for each calendar year and by sex and race and ethnicity among all patients and among those treated with 1 or more classes of antihypertensive medication separately. Age adjustment was performed using direct standardization with the standard population being the KPSC population with hypertension between 2017 and 2019. The age categories used for standardization were 18 to 49 years (14.2%), 50 to 64 years (34.5%), and 65 years or older (51.3%). The age-specific estimated proportion of patients with BP control was calculated in 3 age groups (18-49, 50-64, and ≥65 years) and by sex and race and ethnicity separately. In a sensitivity analysis, the age-adjusted proportion of patients with controlled BP, defined as systolic BP less than 130 mm Hg and diastolic BP less than 80 mm Hg, was estimated. This threshold is similar to the 2017 American College of Cardiology/American Heart Association (ACC/AHA) guideline.^1^

To assess the outcome of QI strategies to reduce the disparity in BP control between Black and White patients, a difference-in-difference analysis was conducted using generalized estimating equation Poisson regression with repeated measurement accounting for correlated outcomes since patients could be included in multiple years. Specifically, the absolute difference in change in BP control before and after program implementation was compared between Black and White patients. The pre-QI period was defined as 2008 and 2009 and the post-QI period was considered 2016 through 2019. The years 2010-2015 were not included to allow for a period between the end of new strategies and the beginning of the assessment period due to the continuous implementation of QI strategies during this time. The analysis was performed among all patients with hypertension and the subgroup of patients using antihypertensive medication.

Among patients with hypertension in 2019, we estimated the association between uncontrolled BP and a priori–selected variables, including age, sex, race and ethnicity, comorbid conditions (ie, CVD, CKD, heart failure, and diabetes), type of health insurance, health care usage, and number of antihypertensive medication classes being taken in the prior year, using multivariable Poisson regression with robust error variance to estimate prevalence ratios.^13^ This analysis was stratified by race and ethnicity and performed among all patients with hypertension in 2019 and the subgroup of patients using antihypertensive medication. All hypothesis tests were 2-sided with a significance level of *P* < .05, and analyses were conducted using SAS Enterprise Guide, version 7.1 (SAS Institute Inc).

## Results

### Demographic Characteristics

The number of KPSC patients with hypertension increased from 624 094 in 2008 (330 551 [53.0%] female patients; 293 543 [47.0%] male patients; 89 407 [14.3%] Black and 284 116 [45.5%] White patients) to 855 257 (444 422 [52.0%] female patients; 410 835 [48.0%] male patients; 107 054 [12.5%] Black and 331 932 [38.8%] White patients) in 2019, parallel to the growth of the overall KPSC population. The mean (SD) age of patients with hypertension increased from 61.8 (13.5) years in 2008 to 64.5 (13.6) years in 2019 (Table 1; eTable 3 in the Supplement). The proportion of patients who self-identified as Black and White decreased from 2008 to 2019. The proportion of patients with CKD decreased while the proportion with heart failure and diabetes increased over time. The proportion of patients with commercial insurance coverage decreased while there was an increase in the proportion of patients with Medicare and Medi-Cal coverage. Black patients had higher mean systolic and diastolic BP compared with White patients in 2008 and 2019 (eTable 4 in the Supplement). The proportion of Black patients taking only 1 class of antihypertensive medication was lower compared with the proportion of White patients in 2008 (21.9% vs 26.2%) and 2019 (23.0% vs 30.1%). The proportion of Black patients taking 3 or more classes of antihypertensive medication decreased from 36.2% in 2008 to 31.9% in 2019, while the proportion of White patients taking 3 or more classes of antihypertensive medication decreased from 29.8% in 2008 to 24.2% in 2019.

**Table 1.** Characteristics of Adults in the Kaiser Permanente Southern California Hypertension Registry, 2008-2009 and 2016-2019

Characteristic	Patients, No. (%)^a^					
	2008	2009	2016	2017	2018	2019
Total adult population, No.	2 412 692	2 414 175	3 313 462	3 766 456	3 897 012	3 960 121
Hypertension population, No. (%)	624 094 (25.9)	644 606 (26.7)	792 066 (23.9)	811 467 (21.5)	838 695 (21.5)	855 257 (21.6)
Age, mean (SD), y	61.8 (13.5)	61.9 (13.5)	64.0 (13.4)	64.1 (13.5)	64.3 (13.5)	64.5 (13.6)
Age group, y						
18-49	112 810 (18.1)	113 932 (17.7)	113 557 (14.3)	115 732 (14.3)	118 632 (14.1)	120 394 (14.1)
50-64	251 886 (40.4)	259 291 (40.2)	282 131 (35.6)	285 437 (35.2)	289 538 (34.5)	290 199 (33.9)
≥65	259 398 (41.6)	271 383 (42.1)	396 378 (50.0)	410 298 (50.6)	430 525 (51.3)	444 664 (52.0)
Sex						
Female	330 551 (53.0)	340 098 (52.8)	413 720 (52.2)	422 897 (52.1)	436 131 (52.0)	444 422 (52.0)
Male	293 543 (47.0)	304 508 (47.2)	378 346 (47.8)	388 570 (47.9)	402 564 (48.0)	410 835 (48.0)
Race and ethnicity^b^						
Asian or Pacific Islander	61 910 (9.9)	66 084 (10.3)	93 589 (11.8)	99 677 (12.3)	104 630 (12.5)	108 117 (12.6)
Black	89 407 (14.3)	92 246 (14.3)	103 003 (13.0)	104 737 (12.9)	106 340 (12.7)	107 054 (12.5)
Hispanic	158 325 (25.4)	167 610 (26.0)	242 376 (30.6)	258 059 (31.8)	271 161 (32.3	281 971 (33.0)
White	284 116 (45.5)	292 183 (45.3)	328 531 (41.5)	329 080 (40.6)	333 359 (39.7)	331 932 (38.8)
Other	30 336 (4.9)	26 483 (4.1)	24 567 (3.1)	19 914 (2.5)	23 205 (2.8)	26 183 (3.1)
Chronic conditions						
Cardiovascular disease	106 390 (17.0)	136 656 (21.2)	138 269 (17.5)	144 062 (17.8)	152 646 (18.2)	158 728 (18.6)
Chronic kidney disease	66 524 (10.7)	57 077 (8.9)	72 496 (9.2)	70 774 (8.7)	73 503 (8.8)	70 168 (8.2)
Heart failure	29 193 (4.7)	34 997 (5.4)	45 872 (5.8)	50 841 (6.3)	56 991 (6.8)	60 282 (7.0)
Diabetes	171 077 (27.4)	189 015 (29.3)	282 714 (35.7)	298 565 (36.8)	308 419 (36.8)	314 950 (36.8)
Type of health insurance						
Commercial	375 090 (60.1)	383 770 (59.5)	393 192 (49.6)	395 353 (48.7)	404 719 (48.3)	410 564 (48.0)
Medicare	222 514 (35.7)	234 469 (36.4)	318 775 (40.2)	328 950 (40.5)	345 382 (41.2)	355 535 (41.6)
Medi-Cal	7 514 (1.2)	8 252 (1.3)	53 614 (6.8)	55 923 (6.9)	57 825 (6.9)	58 862 (6.9)
Private pay	18 976 (3.0)	18 114 (2.8)	26 409 (3.3)	28 249 (3.5)	30 278 (3.6)	29 712 (3.5)
Unknown	0	1 (<0.01)	76 (0.01)	2 992 (0.4)	491 (0.06)	584 (0.07)
SBP, mean (SD), mm Hg	129 (15)	128 (15)	130 (13)	130 (13)	130 (13)	130 (13)
DBP, mean (SD), mm Hg	74 (11)	74 (11)	73 (11)	73 (11)	73 (11)	73 (12)
No. of medications						
0	74 479 (11.9)	78 367 (12.2)	102 745 (13.0)	107 801 (13.3)	108 153 (12.9)	108 633 (12.7)
1	158 947 (25.5)	162 160 (25.2)	231 596 (29.2)	242 345 (29.9)	254 106 (30.3)	262 244 (30.7)
2	198 510 (31.8)	207 079 (32.1)	265 083 (33.5)	269 938 (33.3)	279 152 (33.3)	284 383 (33.3)
≥3	192 158 (30.8)	197 000 (30.6)	192 642 (24.3)	191 351 (23.6)	197 247 (23.5)	199 960 (23.4)
Unknown	0	0	0	32 (0.01)	37 (0.01)	37 (0.01)

Abbreviations: DBP, diastolic blood pressure; SBP, systolic blood pressure.

^a^ Percentages are based on hypertension population.

^b^ Race and ethnicity were self-reported at clinic visits and categorized into mutually exclusive groups, including Hispanic regardless of race and the following non-Hispanic groups: Asian or Pacific Islander, Black, White, and other (Native American or Alaska Native and multiple or other races and ethnicities).

### Age-Adjusted BP Control

Blood pressure control increased an absolute 4.6% (95% CI, 4.3%-4.8%) among Black patients (from 74.8% to 79.4%) and 2.1% (95% CI, 2.0%-2.2%) among White patients (from 80.3% to 82.3%) from before implementation of the QI program to after (difference-in-difference, 2.5%; 95% CI, 2.2%-2.8%) (Table 2 and Figure). Blood pressure control increased an absolute 4.1% (95% CI, 3.8%-4.5%) among Black female patients (from 73.5% to 77.6%) and 1.8% (95% CI, 1.6%-2.0%) among White female patients (from 78.4% to 80.2%) from before implementation of the QI program to after (difference-in-difference, 2.3%; 95% CI, 2.0%-2.7%). The absolute increase in BP control was 5.7% (95% CI, 5.3%-6.1%) among Black male patients (from 75.2% to 80.9%) and 2.4% (95% CI, 2.2%-2.6%) among White male patients (from 82.2% to 84.6%) from before implementation of the QI program to after (difference-in-difference, 3.3%; 95% CI, 2.8%-3.8%). Among all patients, the age-adjusted proportion with BP control increased from 74.3% (95% CI, 74.1%-74.6%) in 2008 to 79.3% (95% CI, 79.1%-79.4%) in 2019 (eTable 5 in the Supplement).

**Table 2.** BP Control Before and After Implementation of a QI Program and Difference-in-Difference Among Black and White Adults With Hypertension at KPSC

Characteristic	Black adults			White adults			Difference-in-difference (95% CI)
	QI BP control		Difference (95% CI)^a^	QI BP control		Difference (95% CI)^a^	
	Before	After		Before	After		
**Among all KPSC patients in the hypertension registry**							
Overall, %	74.8 (74.6-75.1)	79.4 (79.2-79.6)	4.6 (4.3-4.8)	80.3 (80.1-80.4)	82.3 (82.2-82.4)	2.1 (2.0-2.2)	2.5 (2.2-2.8)
Sex, %							
Female	73.5 (73.2-73.8)	77.6 (77.4-77.8)	4.1 (3.8-4.5)	78.4 (78.2-78.6)	80.2 (80.1-80.3)	1.8 (1.6-2.0)	2.3 (2.0-2.7)
Male	75.2 (74.9-75.6)	80.9 (80.7-81.2)	5.7 (5.3-6.1)	82.2 (82.0-82.3)	84.6 (84.4-84.7)	2.4 (2.2-2.6)	3.3 (2.8-3.8)
**Among all KPSC patients in the hypertension registry treated with antihypertensive medications**							
Overall, %	76.0 (75.7-76.2)	81.4 (81.2-81.6)	5.4 (5.2-5.7)	81.1 (80.9-81.2)	83.9 (83.8-84.1)	2.9 (2.7-3.0)	2.5 (2.2-2.8)
Sex, %							
Female	74.0 (73.7-74.3)	78.9 (78.7-79.1)	4.9 (4.5-5.2)	78.7 (78.5-78.9)	81.3 (81.1-81.4)	2.6 (2.4-2.8)	2.3 (1.9-2.7)
Male	76.2 (75.8-76.6)	83.0 (82.7-83.2)	6.8 (6.3-7.2)	82.7 (82.5-82.9)	85.9 (85.8-86.1)	3..2 (1.9-2.7)	3.5 (3.1-4.0)

Abbreviations: BP, blood pressure; KPSC, Kaiser Permanente Southern California; QI, quality improvement.

^a^ Difference indicates the value from the post-QI period (2016-2019) minus the value from the pre-QI period (2008-2009).

**Figure.**
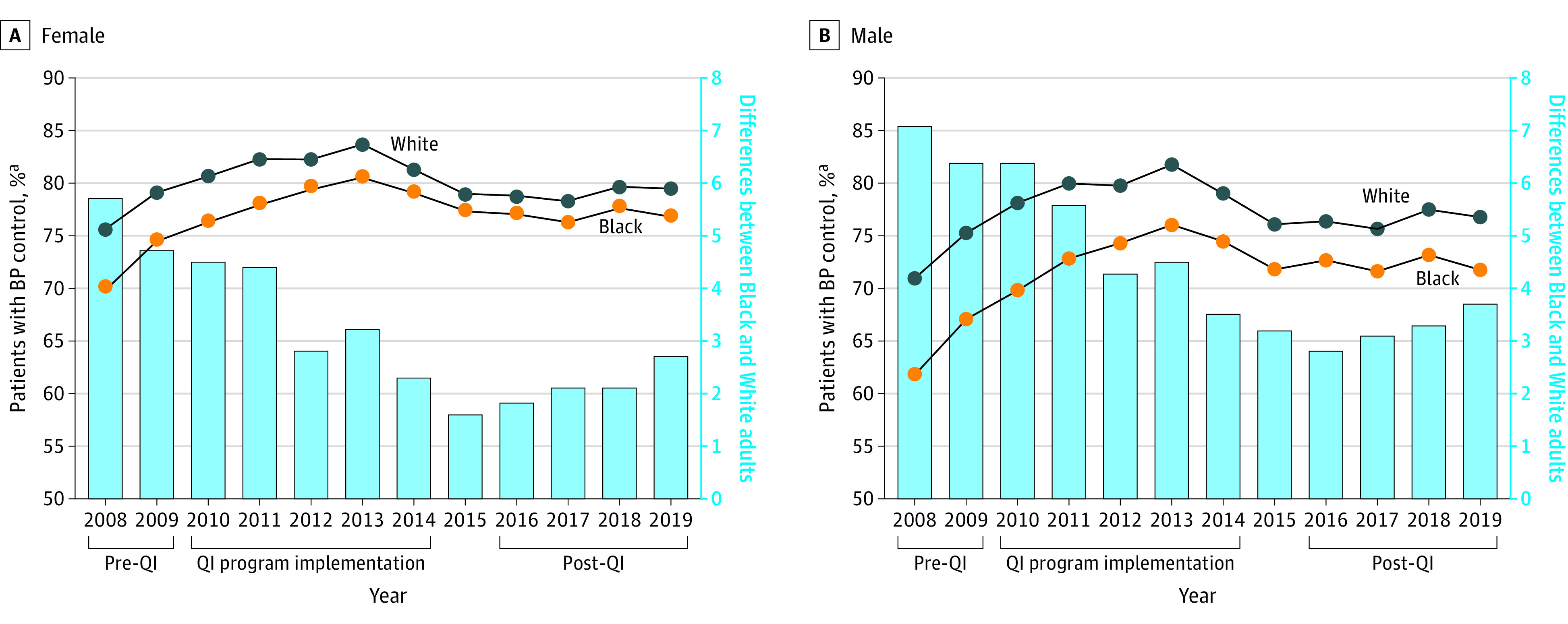
Age-Adjusted Proportion of Patients With Blood Pressure (BP) Control by Sex and Race, 2008-2019 Blood pressure control in female (A) and male (B) adults was defined as systolic BP less than 140 mm Hg and diastolic BP less than 90 mm Hg. QI indicates quality improvement.

The absolute difference in BP control post-QI implementation vs pre-QI between Black and White patients using antihypertensive medication was 2.5% (95% CI, 2.2%-2.8%) (Table 2). The absolute difference in BP control post-QI implementation vs pre-QI between Black and White individuals was 2.3% (95% CI, 1.9%-2.7%) for female patients and 3.5% (95% CI, 3.1%-4.0%) for male patients.

The age-adjusted proportion of Black patients using antihypertensive medication with BP control in 2008 was 70.5% (95% CI, 69.9%-71.1%) and the age-adjusted proportion with BP control among White patients using antihypertensive medication was 76.3% (95% CI, 76.0%-76.7%) (eFigure 1 and eTable 6 in the Supplement). In 2019, the age-adjusted proportion of Black patients using antihypertensive medication with BP control was 78.4% (95% CI, 77.8%-79.0%), and the age-adjusted proportion of White patients was 81.2% (95% CI, 80.9%-81.5%). The absolute difference in BP control between treated Black and White female patients decreased from 5.4% in 2008 to 2.5% in 2019. The absolute difference in BP control between treated Black and White male patients decreased from 6.7% in 2008 to 3.0% in 2019.

### Age-Specific BP Control

The largest reduction in the BP control disparity between Black and White female patients was among those aged 50 to 64 years (difference-in-difference, 3.8%; 95% CI, 3.2%-4.4%) (Table 3). Among Black and White male patients, those aged 18 to 49 years had the largest reduction in BP control disparity (difference-in-difference, 4.2%; 95% CI, 3.0%-5.5%). Black male patients who were 65 years or older had the largest absolute increase in BP control from the pre-QI implementation period to the post-QI period (7.3%; 95% CI, 6.7%-7.9%) compared with Black and White patients in the other age groups. Black male patients aged 18 to 49 years had the lowest proportion of BP control each year compared with male and female patients in other age and racial and ethnic groups, with 58.3% in 2008 and 63.0% in 2019 (eFigure 2 and eTable 7 in the Supplement).

**Table 3.** Difference in BP Control After vs Before Implementation of a QI Program Stratified by Age Group Among Adults in the Kaiser Permanente Southern California Hypertension Registry

Characteristic	Female patients, age, y			Male patients, age, y		
	18-49	50-64	≥65	18-49	50-64	≥65
**Among all patients in the hypertension registry**						
Difference in BP control post-QI vs pre-QI, %^a^						
Black	0.4 (−0.4 to 1.2)	4.3 (3.8 to 4.9)	5.6 (5.1 to 6.1)	3.8 (2.7 to 4.9)	5.5 (4.8 to 6.1)	7.3 (6.7 to 7.9)
White	−3.1 (−3.7 to −2.4)	0.5 (0.2 to 0.9)	3.4 (3.2 to 3.7)	−0.4 (−1.1 to 0.2)	2.0 (1.7 to 2.4)	4.0 (3.7 to 4.2)
Absolute difference-in-difference between Black and White	3.5 (2.4 to 4.5)	3.8 (3.2 to 4.4)	2.2 (1.6 to 2.8)	4.2 (3.0 to 5.5)	3.4 (2.7 to 4.2)	3.3 (2.7 to 4.0)
**Among all patients in the hypertension registry treated with antihypertensive medications**						
Difference in BP control post-QI vs pre-QI, %^a^						
Black	1.1 (0.2 to 1.9)	4.9 (4.4 to 5.5)	6.3 (5.7 to 6.8)	5.4 (4.3 to 6.6)	6.1 (5.4 to 6.8)	8.1 (7.5 to 8.8)
White	−2.1 (−2.9 to −1.4)	1.3 (1.0 to 1.7)	4.0 (3.7 to 4.3)	0.7 (0.0 to 1.4)	2.9 (2.5 to 3.2)	4.5 (4.2 to 4.7)
Absolute difference-in-difference between Black and White	3.2 (2.0 to 4.3)	3.6 (2.9 to 4.2)	2.3 (1.7 to 2.8)	4.7 (3.4 to 6.1)	3.2 (2.4 to 4.0)	3.7 (3.0 to 4.4)

Abbreviations: BP, blood pressure; QI, quality improvement.

^a^ Difference indicates the value from the post-QI period (2016-2019) minus that from the pre-QI period (2008-2009).

### BP Control Defined by the 2017 ACC/AHA BP Guideline

In the sensitivity analysis, the age-adjusted proportion of patients with BP control defined by the 2017 ACC/AHA BP guideline (<130/80 mm Hg) among Black patients in 2008 was 36.8% (95% CI, 36.4%-37.2%) and, among White patients, 42.7% (95% CI, 42.5%-43.0%) (eFigure 3 and eTable 8 in the Supplement). In 2019, the proportion of patients with BP control was 33.8% (95% CI, 33.5%-34.2%) among Black patients and 38.3% (95% CI, 38.0%-38.5%) among White patients. The difference in BP control defined by the 2017 ACC/AHA BP guideline between Black and White patients was 5.9% in 2008 and 4.5% in 2019.

### Factors Associated With Uncontrolled BP

After multivariable adjustment, uncontrolled BP was more common among Black than White patients overall (prevalence ratio, 1.13; 95% CI, 1.12-1.14) and among those treated with antihypertensive medication (prevalence ratio, 1.12; 95% CI, 1.10-1.13) (Table 4). Patients who were aged 50 to 64 years and 65 years or older were less likely than those aged 18 to 49 years to have uncontrolled BP among all adult patients with hypertension and among those taking antihypertensive medication. Female patients were more likely than male patients to have uncontrolled BP in both the overall population with hypertension and among those taking antihypertensive medication. Chronic kidney disease was associated with a higher prevalence of uncontrolled BP among the overall and treated patient groups. Patients with 13 or more vs no outpatient visits in 2018 had a lower likelihood of uncontrolled BP in the overall and treated patient groups. Among all patients with hypertension, being treated with 1, 2, and 3 or more classes of antihypertensive medication was associated with a lower likelihood of uncontrolled BP compared with patients not treated with medication. Among patients treated with antihypertensive medication, those treated with 3 or more vs 1 medication class were more likely to have uncontrolled BP. Multivariate results stratified by race and ethnicity are presented in eTable 9 and eTable 10 in the Supplement.

**Table 4.** Factors Associated With Uncontrolled Blood Pressure Among Kaiser Permanente Southern California Adults With Hypertension, Overall and Treated With Antihypertensive Medication, 2019

Characteristic	Prevalence ratio (95% CI)^a^	
	Overall	Treated with antihypertensives
No. of patients	855 257	746 624
Age group, y		
18-49	1 [Reference]	1 [Reference]
50-64	0.80 (0.79-0.81)	0.76 (0.75-0.77)
≥65	0.75 (0.73-0.76)	0.68 (0.66-0.69)
Sex		
Male	1 [Reference]	1 [Reference]
Female	1.06 (1.05-1.07)	1.09 (1.08-1.10)
Race		
Black	1.13 (1.12-1.14)	1.12 (1.10-1.13)
White	1 [Reference]	1 [Reference]
Chronic conditions		
Cardiovascular disease	0.98 (0.97-0.99)	0.99 (0.98-1.00)
Chronic kidney disease	1.27 (1.25-1.29)	1.29 (1.27-1.31)
Heart failure	0.99 (0.97-1.01)	0.97 (0.95-0.99)
Diabetes	0.96 (0.95-0.97)	0.96 (0.95-0.97)
Insurance		
Commercial	1 [Reference]	1 [Reference]
Medicare	0.84 (0.83-0.86)	0.85 (0.84-0.86)
Medi-Cal	0.92 (0.90-0.93)	0.92 (0.90-0.94)
Private pay	0.94 (0.92-0.96)	0.94 (0.92-0.96)
No. of outpatient visits^b^		
0	1 [Reference]	1 [Reference]
1-6	0.74 (0.73-0.74)	0.88 (0.87-0.89)
7-12	0.53 (0.52-0.53)	0.65 (0.64-0.67)
≥13	0.46 (0.46-0.47)	0.59 (0.58-0.60)
No. of inpatient visits^b^		
0	1 [Reference]	1 [Reference]
1-3	1.02 (1.00-1.03)	1.03 (1.01-1.04)
≥3	0.92 (0.88-0.96)	0.94 (0.90-0.98)
No. of antihypertensive medications		
0	1 [Reference]	NA
1	0.61 (0.60-0.62)	1 [Reference]
2	0.61 (0.61-0.62)	1.01 (1.00-1.02)
≥3	0.69 (0.68-0.70)	1.13 (1.12-1.14)

Abbreviation: NA, not applicable.

^a^ Adjusted for all variables in the table.

^b^ Visits in 2018.

## Discussion

The estimated proportion of KPSC patients with hypertension who had controlled BP increased from 2008 through 2019. The disparity in BP control between Black and White patients was reduced although not eliminated following the implementation of a QI program aimed at reducing disparities in BP control between Black and White patients. The largest reductions in the BP control disparity between Black and White patients were among female patients aged 50 to 64 years and male patients aged 18 to 49 years.

The persistent gap in BP control between Black and White patients in the current study, particularly in the younger age groups, has been reported in other populations.^4,5,14^ Hardy and colleagues^15^ analyzed National Health and Nutrition Examination Survey (NHANES) data from 1999-2002 and 2015-2018 and found a continued disparity in BP control between Black and White adults. An analysis of NHANES data comparing the periods 2009-2014 and 2015-2018 found that differences in BP control between Black and White adults were reduced when adjusted for modifiable risk factors, including educational level, obesity, and access to care.^16^ We did not assess those factors in the current study; however, having more outpatient visits was associated with a lower risk of uncontrolled BP, suggesting that more health care usage may provide a benefit to controlling BP.

In the current study, a similar proportion of Black and White patients with hypertension were treated with antihypertensive medication. A higher proportion of Black than White patients were treated with 3 or more antihypertensive medications; however, the prevalence of uncontrolled BP was higher in Black than in White patients throughout the study period, with the lowest BP control among Black individuals aged 18 to 49 years. In an NHANES analysis from 2011-2018, Lu and colleagues^17^ found that Black adults had a similar treatment rate, received more intensive antihypertensive therapy if treated, and had lower BP control compared with White adults. Lower medication adherence, environmental issues, and lifestyle factors may be contributors to more intensive treatment yet lower BP control among Black patients.^18,19,20^

Throughout the study period, the proportion of KPSC patients with BP control was higher than the proportion among the general US population.^5^ The KPSC QI strategies around BP control, the setting of an integrated health care system, a preferred medication algorithm, and no-copay BP checks may have contributed to an improvement in BP control. The increase in BP control among Black and White patients from 2008 to 2013 followed by a decrease in later years is consistent with findings observed in the overall US population.^5^ These findings may be a result of a higher BP goal for patients 60 years or older, which was issued in the Eighth Joint National Committee’s 2014 report^21^ on the management of high BP in adults, thus potentially resulting in less antihypertensive medication use and lower BP control rates.^22^

Although many of the strategies implemented to address BP control within the KPSC population are relevant across the life span, tailored interventions may be required to further reduce the disparity in BP control between younger Black and White patients. For example, the Los Angeles Barbershop Blood Pressure Study enrolled Black male patrons aged 35 to 79 years with uncontrolled BP and used clinical pharmacists combined with barber education for hypertension management.^23^ The intervention not only substantially reduced mean systolic BP after 6 months but was also shown to be highly cost-effective for reducing cardiovascular morbidity and mortality in Black men.^24^ By partnering with respected community leaders to create health education campaigns for social media and television, health care organizations could potentially improve trust in the health care system among Black patients while also increasing awareness of strategies to manage BP and to prevent hypertension and CVD outcomes. In addition, the Centers for Disease Control and Prevention^25^ and the US Surgeon General’s office^26^ have published resources for improving hypertension awareness, treatment, and control across diverse settings in the US.

### Strengths and Limitations

A strength of this study is that it was conducted in a large, diverse population using electronic health record and administrative data, which provide complete information about patients at the point of care compared with solely using administrative claims data. In addition, trends are reported over a 12-year period. However, this study has limitations. Interpretation of the results requires several considerations. The data were limited to a single health plan; therefore, the findings may not be generalizable to uninsured populations or those with different demographic characteristics. Consistent with the KPSC hypertension registry methods, we used the last BP measurement in each calendar year to define BP control; however, this approach may not fully capture a patient’s BP control status during other time points in the year. In addition, patients who did not have a hospitalization or an outpatient visit in the calendar year may have had undiagnosed elevated BP, thereby possibly underestimating the prevalence of hypertension.

## Conclusions

This QI study found that reductions in BP control disparities between Black and White patients occurred within a large integrated health care system that implemented QI strategies aimed at reducing disparities. However, disparities persisted and were largest among younger age groups. These findings suggest more focused interventions may be needed to increase BP control among Black patients even in an integrated health care setting.

## Notes

Supplement 1. **eTable 1.** Timeline of Health Care Delivery Changes, Kaiser Permanente Southern California, 2009-2014 **eTable 2.** *International Classification of Diseases* (*ICD*) Diagnosis Codes for Primary and Secondary Hypertension and Hypertension Combination Codes **eTable 3.** Characteristics of All Adults in the Kaiser Permanente Southern California Hypertension Registry, 2008-2019 **eTable 4.** Characteristics of Adults in the Kaiser Permanente Southern California Hypertension Registry by Race and Ethnicity in 2008 and 2019 **eTable 5.** Age-Adjusted Proportion of Patients With Blood Pressure Control Among Adults in the Kaiser Permanente Southern California Hypertension Registry, 2008-2019 **eTable 6.** Age-Adjusted Proportion of Patients With Blood Pressure Control Among Adults in the Kaiser Permanente Southern California Hypertension Registry and Treated With Antihypertensive Medication, 2008-2019 **eFigure 1.** Age-Adjusted Proportion of Patients With Blood Pressure Control by Sex and Race and Ethnicity Among Adults in the Kaiser Permanente Southern California Hypertension Registry and Treated With Antihypertensive Medication, 2008-2019 **eTable 7.** Age-Specific Proportion of Patients With Blood Pressure Control by Sex and Race and Ethnicity Among Adults in the Kaiser Permanente Southern California Hypertension Registry, 2008-2019 **eFigure 2.** Age-Specific Proportion of Patients With Blood Pressure Control by Sex and Race and Ethnicity Among Adults in the Kaiser Permanente Southern California Hypertension Registry, 2008-2019 **eTable 8.** Age-Adjusted Proportion of Patients With Blood Pressure Control (<130/80 mm Hg) by Race and Ethnicity Among Adults in the Kaiser Permanente Southern California Hypertension Registry, 2008-2019 **eTable 9.** Factors Associated With Uncontrolled Blood Pressure by Race and Ethnicity Among Kaiser Permanente Southern California Adults in the Hypertension Registry in 2019 **eTable 10.** Factors Associated With Uncontrolled Blood Pressure by Race and Ethnicity Among Adults in the Kaiser Permanente Southern California Hypertension Registry and Treated With Antihypertensive Medication in 2019 **eFigure 3.** Age-Adjusted Proportion of Patients With Blood Pressure Control (<130/80 mm Hg) by Race and Ethnicity Among Adults in the Kaiser Permanente Southern California Hypertension Registry, 2008-2019

Supplement 2.
Data Sharing Statement
