# Supplementary material for: Blood Pressure Control Among Black and White Adults Following a Quality Improvement Program in a Large Integrated Health System
Source: JAMA Netw Open. 2023 Jan 6;6(1):e2249930. doi: 10.1001/jamanetworkopen.2022.49930 (PMC9856959; doi:10.1001/jamanetworkopen.2022.49930)
Supplement: Supplement 1. — eTable 1. Timeline of Health Care Delivery Changes, Kaiser Permanente Southern California, 2009-2014 eTable 2. International Classification of Diseases (ICD) Diagnosis Codes for Primary and Secondary Hypertension and Hypertension Combination Codes eTable 3. Characteristics of All Adults in the Kaiser Permanente Southern California Hypertension Registry, 2008-2019 eTable 4. Characteristics of Adults in the Kaiser Permanente Southern California Hypertension Registry by Race and Ethnicity in 2008 and 2019 eTable 5. Age-Adjusted Proportion of Patients With Blood Pressure Control Among Adults in the Kaiser Permanente Southern California Hypertension Registry, 2008-2019 eTable 6. Age-Adjusted Proportion of Patients With Blood Pressure Control Among Adults in the Kaiser Permanente Southern California Hypertension Registry and Treated With Antihypertensive Medication, 2008-2019 eFigure 1. Age-Adjusted Proportion of Patients With Blood Pressure Control by Sex and Race and Ethnicity Among Adults in the Kaiser Permanente Southern California Hypertension Registry and Treated With Antihypertensive Medication, 2008-2019 eTable 7. Age-Specific Proportion of Patients With Blood Pressure Control by Sex and Race and Ethnicity Among Adults in the Kaiser Permanente Southern California Hypertension Registry, 2008-2019 eFigure 2. Age-Specific Proportion of Patients With Blood Pressure Control by Sex and Race and Ethnicity Among Adults in the Kaiser Permanente Southern California Hypertension Registry, 2008-2019 eTable 8. Age-Adjusted Proportion of Patients With Blood Pressure Control (<130/80 mm Hg) by Race and Ethnicity Among Adults in the Kaiser Permanente Southern California Hypertension Registry, 2008-2019 eTable 9. Factors Associated With Uncontrolled Blood Pressure by Race and Ethnicity Among Kaiser Permanente Southern California Adults in the Hypertension Registry in 2019 eTable 10. Factors Associated With Uncontrolled Blood Pressure by Race and Ethnicity Among Adults in the Kaise [file jamanetwopen-e2249930-s001.pdf]

Supplementary Online Content

Harrison TN, Zhou H, Wei R, et al. Blood pressure control among Black and White adults following a quality improvement program in a large integrated health system. *JAMA Netw Open*. 2023;6(1):e2249930. doi:10.1001/jamanetworkopen.2022.49930

eTable 1. Timeline of Health Care Delivery Changes, Kaiser Permanente Southern California, 2009-2014

eTable 2. *International Classification of Diseases* (*ICD*) Diagnosis Codes for Primary and Secondary Hypertension and Hypertension Combination Codes

eTable 3. Characteristics of All Adults in the Kaiser Permanente Southern California Hypertension Registry, 2008-2019

eTable 4. Characteristics of Adults in the Kaiser Permanente Southern California Hypertension Registry by Race and Ethnicity in 2008 and 2019

eTable 5. Age-Adjusted Proportion of Patients With Blood Pressure Control Among Adults in the Kaiser Permanente Southern California Hypertension Registry, 2008-2019

eTable 6. Age-Adjusted Proportion of Patients With Blood Pressure Control Among Adults in the Kaiser Permanente Southern California Hypertension Registry and Treated With Antihypertensive Medication, 2008-2019

eFigure 1. Age-Adjusted Proportion of Patients With Blood Pressure Control by Sex and Race and Ethnicity Among Adults in the Kaiser Permanente Southern California Hypertension Registry and Treated With Antihypertensive Medication, 2008-2019

eTable 7. Age-Specific Proportion of Patients With Blood Pressure Control by Sex and Race and Ethnicity Among Adults in the Kaiser Permanente Southern California Hypertension Registry, 2008-2019

eFigure 2. Age-Specific Proportion of Patients With Blood Pressure Control by Sex and Race and Ethnicity Among Adults in the Kaiser Permanente Southern California Hypertension Registry, 2008-2019

eTable 8. Age-Adjusted Proportion of Patients With Blood Pressure Control (<130/80 mm Hg) by Race and Ethnicity Among Adults in the Kaiser Permanente Southern California Hypertension Registry, 2008-2019

eTable 9. Factors Associated With Uncontrolled Blood Pressure by Race and Ethnicity Among Kaiser Permanente Southern California Adults in the Hypertension Registry in 2019

eTable 10. Factors Associated With Uncontrolled Blood Pressure by Race and Ethnicity Among Adults in the Kaiser Permanente Southern California Hypertension Registry and Treated With Antihypertensive Medication in 2019

eFigure 3. Age-Adjusted Proportion of Patients With Blood Pressure Control (<130/80 mm Hg) by Race and Ethnicity Among Adults in the Kaiser Permanente Southern California Hypertension Registry, 2008-2019

This supplementary material has been provided by the authors to give readers additional information about their work.

© 2023 Harrison TN et al. *JAMA Network Open.*

eTable 1. Timeline of Health Care Delivery Changes, Kaiser Permanente Southern California, 2009-2014

Year	Health Care Delivery Changes
2009	• Developed national and regional equitable care reports with Healthcare Effectiveness Data Information Set (HEDIS) measures stratified by race and ethnicity to guide disparity reduction strategies
2010	• Began implementation of the Equitable Care Health Outcomes (ECHO)[a] program to reduce the disparity in hypertension control<br>• Performance feedback provided monthly to physicians with physician-specific performance data on blood pressure control rates for non-Hispanic Black and non-Hispanic White patients
2011	• Established collaboration between regional hypertension team and cultural diversity committee
2012	• First annual Kaiser National Quality Conference presentation on hypertension disparity closure<br>• Tailored outreach to non-Hispanic Black patients with uncontrolled blood pressure via letters with physician photos and voice recordings by their primary care provider<br>• Panel management programs for non-Hispanic Black patients naïve to thiazides or on suboptimal lisinopril-hydrochlorothiazide doses
2013	• Introduced clinician and staff education programs around building trust and integrating culturally tailored communication tools including use of the Acknowledge, Introduce, Duration, Explanation (AIDET) communication model[b]<br>• Began administering salt questionnaire to assess dietary sodium intake and counsel patients on measures to reduce salt consumption<br>• Introduced comprehensive program with treatment intensification tailored to individual patients<br>• Produced African American storytelling videos with Kaiser Permanente patients distributed to all regional medical centers for viewing in waiting rooms and provided to patients with uncontrolled blood pressure[c]<br>• Introduced large group appointments at participating medical centers
2014	• Expanded use of ECHO communication tools and best practices throughout the Kaiser Permanente Southern California region

[a] Bartolome R. Equitable Care Health Outcomes: ECHO. ECHO toolkit. Hypertension management for African Americans [Internet]. Oakland, CA: Kaiser Permanente; 2012 [cited 2022 Oct21]. Available from: https://wiki.kp.org/wiki/display/equitablecarenatl/Hypertension?preview=/181409536/188847408/ECHO20Toolkit20020813 20v7.pdf
[b] https://www.studergroup.com/aidet
[c] Houston TK, et al. Culturally appropriate storytelling to improve blood pressure: a randomized trial. Ann Intern Med. 2011;154(2):77-84.

© 2023 Harrison TN et al. *JAMA Network Open.*

eTable 2. ***International Classification of Diseases*** **(*ICD*) Diagnosis Codes for Primary and Secondary Hypertension and Hypertension Combination Codes**

ICD-9 Codes	227.0, 250.40, 250.41, 255.9, 362.11, 401.xx, 402.xx, 403.xx, 405.xx, 428, 585.x, 587, 642.2x, 779.89, 996.81, V45.11, V65.3, E933.1
ICD-10 Codes	D35.00, E10.22, E11.22, E11.29, E27.9, E34.9, H35.03x, I10, I11.x, I12.x, I13.x, I15.x, I16.x, I70.1, I77.89, O10.1x, O10.2x, O10.3x, P29.2, N08, N18.x, T86.19, Z71.3, Z99.2

© 2023 Harrison TN et al. *JAMA Network Open.*

eTable 3. Characteristics of All Adults in the Kaiser Permanente Southern California Hypertension Registry, 2008-2019

**Characteristic**	**2008**	**2009**	**2010**	**2011**	**2012**	**2013**
**Total adult population, N**	2,412,692	2,414,175	2,471,322	2,606,503	2,688,011	2,746,327
**Hypertension population, N, (%)**	624,094 (25.9)	644,606 (26.7)	658,929 (26.7)	673,412 (25.8)	696,282 (25.9)	714,573 (26.0)
**Age, mean (SD)**	61.8 (13.5)	61.9 (13.5)	62.4 (13.4)	62.7 (13.5)	63.1 (13.4)	63.4 (13.4)
**Age group, y**						
18-49	112,810 (18.1)	113,932 (17.7)	110,041 (16.7)	109,360 (16.2)	108,347 (15.6)	106,837 (15.0)
50-64	251,886 (40.4)	259,291 (40.2)	263,194 (39.9)	263,212 (39.1)	263,471 (37.8)	264,391 (37.0)
65+	259,398 (41.6)	271,383 (42.1)	285,694 (43.4)	300,840 (44.7)	324,464 (46.6)	343,345 (48.0)
**Sex**						
Female	330,551 (53.0)	340,098 (52.8)	346,496 (52.6)	354,549 (52.6)	365,773 (52.5)	374,307 (52.4)
Male	293,543 (47.0)	304,508 (47.2)	312,433 (47.4)	318,863 (47.4)	330,509 (47.5)	340,266 (47.6)
**Race/ethnicity**						
API	61,910 (9.9)	66,084 (10.3)	69,600 (10.6)	73,513 (10.9)	77,128 (11.1)	80,667 (11.3)
Hispanic	158,325 (25.4)	167,610 (26.0)	174,826 (26.5)	183,015 (27.2)	193,420 (27.8)	202,614 (28.4)
NHB	89,407 (14.3)	92,246 (14.3)	93,427 (14.2)	94,759 (14.1)	96,703 (13.9)	97,925 (13.7)
NHW	284,116 (45.5)	292,183 (45.3)	299,125 (45.4)	303,939 (45.1)	311,070 (44.7)	314,944 (44.1)
Other	30,336 (4.9)	26,483 (4.1)	21,951 (3.3)	18,186 (2.7)	17,961 (2.6)	18,423 (2.6)
**Chronic Conditions**						
Cardiovascular disease	106,390 (17.0)	136,656 (21.2)	134,825 (20.5)	125,487 (18.6)	131,648 (18.9)	133,833 (18.7)
Chronic kidney disease	66,524 (10.7)	57,077 (8.9)	60,223 (9.1)	60,523 (9.0)	67,578 (9.7)	67,720 (9.5)
Heart failure	29,193 (4.7)	34,997 (5.4)	35,970 (5.5)	36,038 (5.4)	36,384 (5.2)	36,718 (5.1)
Diabetes	171,077 (27.4)	189,015 (29.3)	197,319 (29.9)	206,745 (30.7)	219,723 (31.6)	230,808 (32.3)
**Type of health insurance**						
Commercial	375,090 (60.1)	383,770 (59.5)	384,244 (58.3)	386,208 (57.4)	389,312 (55.9)	390,812 (54.7)
Medicare	222,514 (35.7)	234,469 (36.4)	248,755 (37.8)	261,420 (38.8)	281,262 (40.4)	298,088 (41.7)
Medi-Cal	7,514 (1.2)	8,252 (1.3)	8,654 (1.3)	9,181 (1.4)	9,993 (1.4)	10,831 (1.5)
Private pay	18,976 (3.0)	18,114 (2.8)	17,276 (2.6)	16,589 (2.5)	15,714 (2.3)	14,841 (2.1)
Unknown	0 (0.0)	1 (0.0)	0 (0.0)	14 (0.0)	1 (0.0)	1 (0.0)
**SBP, mean (SD), mm Hg**	129 (15)	128 (15)	128 (14)	128 (14)	128 (14)	128 (13)

© 2023 Harrison TN et al. *JAMA Network Open.*

**DBP, mean (SD), mm Hg**	74 (11)	74 (11)	73 (11)	73 (11)	73 (11)	73 (11)
**No. of Medications**						
0	74,479 (11.9)	78,367 (12.2)	78,458 (11.9)	78,821 (11.7)	83,917 (12.1)	87,267 (12.2)
1	158,947 (25.5)	162,160 (25.2)	166,961 (25.3)	172,869 (25.7)	182,670 (26.2)	190,716 (26.7)
2	198,510 (31.8)	207,079 (32.1)	215,100 (32.6)	223,952 (33.3)	233,706 (33.6)	240,573 (33.7)
≥ 3	192,158 (30.8)	197,000 (30.6)	198,410 (30.1)	197,770 (29.4)	195,989 (28.1)	196,017 (27.4)
Unknown	0 (0.0)	0 (0.0)	0 (0.0)	0 (0.0)	0 (0.0)	0 (0.0)

Data are expressed as N (%) unless otherwise indicated. Percentages are based on hypertension population
API = Asian/Pacific Islander; NHB = Non-Hispanic Black; NHW = Non-Hispanic White; Other = Native American/Alaskan Native and multiple or other races and ethnicities
SBP = systolic blood pressure; DBP = diastolic blood pressure

© 2023 Harrison TN et al. *JAMA Network Open.*

eTable 3. Characteristics of All Adults in the Kaiser Permanente Southern California Hypertension Registry, 2008-2019 (continued)

Characteristic	2014	2015	2016	2017	2018	2019
**Total adult population (N)**	2,906,270	3,182,140	3,313,462	3,766,456	3,897,012	3,960,121
**Hypertension population (N)**	744,950 (26.6)	770,646 (24.3)	792,066 (23.9)	811,467 (21.5)	838,695 (21.5)	855,257 (21.6)
**Age, mean (SD)**	63.7 (13.4)	63.8 (13.4)	64.0 (13.4)	64.1 (13.5)	64.3 (13.5)	64.5 (13.6)
**Age group, y**						
18-49	108,755 (14.6)	111,341 (14.4)	113,557 (14.3)	115,732 (14.3)	118,632 (14.1)	120,394 (14.1)
50-64	271,578 (36.5)	279,282 (36.2)	282,131 (35.6)	285,437 (35.2)	289,538 (34.5)	290,199 (33.9)
65+	364,617 (48.9)	380,023 (49.3)	396,378 (50.0)	410,298 (50.6)	430,525 (51.3)	444,664 (52.0)
**Sex**						
Female	390,770 (52.5)	403,180 (52.3)	413,720 (52.2)	422,897 (52.1)	436,131 (52.0)	444,422 (52.0)
Male	354,180 (47.5)	367,466 (47.7)	378,346 (47.8)	388,570 (47.9)	402,564 (48.0)	410,835 (48.0)
**Race/ethnicity**						
API	85,115 (11.4)	89,659 (11.6)	93,589 (11.8)	99,677 (12.3)	104,630 (12.5)	108,117 (12.6)
Hispanic	218,041 (29.3)	230,882 (30.0)	242,376 (30.6)	258,059 (31.8)	271,161 (32.3	281,971 (33.0)
NHB	100,828 (13.5)	102,080 (13.2)	103,003 (13.0)	104,737 (12.9)	106,340 (12.7)	107,054 (12.5)
NHW	321,277 (43.1)	326,162 (42.3)	328,531 (41.5)	329,080 (40.6)	333,359 (39.7)	331,932 (38.8)
Other	19,689 (2.6)	21,863 (2.8)	24,567 (3.1)	19,914 (2.5)	23,205 (2.8)	26,183 (3.1)
**Chronic Conditions**						
Cardiovascular disease	139,043 (18.7)	140,389 (18.2)	138,269 (17.5)	144,062 (17.8)	152,646 (18.2)	158,728 (18.6)
Chronic kidney disease	70,806 (9.5)	66,773 (8.7)	72,496 (9.2)	70,774 (8.7)	73,503 (8.8)	70,168 (8.2)
Heart failure	38,230 (5.1)	40,300 (5.2)	45,872 (5.8)	50,841 (6.3)	56,991 (6.8)	60,282 (7.0)
Diabetes	249,930 (33.5)	263,830 (34.2)	282,714 (35.7)	298,565 (36.8)	308,419 (36.8)	314,950 (36.8)
**Type of health insurance**						
Commercial	389,070 (52.2)	388,434 (50.4)	393,192 (49.6)	395,353 (48.7)	404,719 (48.3)	410,564 (48.0)
Medicare	316,352 (42.5)	309,472 (40.2)	318,775 (40.2)	328,950 (40.5)	345,382 (41.2)	355,535 (41.6)
Medi-Cal	19,371 (2.6)	45,362 (5.9)	53,614 (6.8)	55,923 (6.9)	57,825 (6.9)	58,862 (6.9)
Private pay	20,108 (2.7)	27,356 (3.5)	26,409 (3.3)	28,249 (3.5)	30,278 (3.6)	29,712 (3.5)
Unknown	49 (0.0)	22 (0.0)	76 (0.0)	2992 (0.4)	491 (0.1)	584 (0.1)
**SBP, mean (SD), mm Hg**	128 (13)	129 (13)	130 (13)	130 (13)	130 (13)	130 (13)

© 2023 Harrison TN et al. *JAMA Network Open.*

**DBP, mean (SD), mm Hg**	73 (11)	73 (11)	73 (11)	73 (11)	73 (11)	73 (12)
**No. of Medications**						
0	94,512 (12.7)	100,982 (13.1)	102,745 (13.0)	107,801 (13.3)	108,153 (12.9)	108,633 (12.7)
1	204,293 (27.4)	220,041 (28.6)	231,596 (29.2)	242,345 (29.9)	254,106 (30.3)	262,244 (30.7)
2	249,974 (33.6)	258,437 (33.5)	265,083 (33.5)	269,938 (33.3)	279,152 (33.3)	284,383 (33.3)
≥ 3	196,171 (26.3)	191,186 (24.8)	192,642 (24.3)	191,351 (23.6)	197,247 (23.5)	199,960 (23.4)
Unknown	0 (0.0)	0 (0.0)	0 (0.0)	32 (0.0)	37 (0.0)	37 (0.0)

Data are expressed as N (%) unless otherwise indicated. Percentages are based on hypertension population.
API = Asian/Pacific Islander; NHB = Non-Hispanic Black; NHW = Non-Hispanic White; Other = Native American/Alaskan Native and multiple or other races and ethnicities
SBP = systolic blood pressure; DBP = diastolic blood pressure

© 2023 Harrison TN et al. *JAMA Network Open.*

eTable 4. Characteristics of Adults in the Kaiser Permanente Southern California Hypertension Registry by Race and Ethnicity in 2008 and 2019

	2008					2019			
	Race/ethnicity					Race/ethnicity			
	NHW (N=284,116)	NHB (N=89,407)	Hispanic (N=158,325)	API (N=61,910)		NHW (N=331,932)	NHB (N=107,054)	Hispanic (N=281,971)	API (N=108,117)
**Age, mean (SD)**	65.1 (13.2)	60.0 (13.2)	58.4 (13.3)	60.5 (12.3)		67.5 (12.9)	63.7 (13.3)	61.8 (13.8)	64.0 (13.2)
**Age group, y**									
18-49	33,122 (11.7)	19,883 (22.2)	40,076 (25.3)	10,816 (17.5)		29,642 (8.9)	15,090 (14.1)	53,792 (19.1)	16,012 (14.8)
50-64	105,035 (37.0)	36,408 (40.7)	67,128 (42.4)	28,452 (46.0)		98,692 (29.7)	40,211 (37.6)	104,101 (36.9)	36,548 (33.8)
65+	145,959 (51.4)	33,116 (37.0)	51,121 (32.3)	22,642 (36.6)		203,598 (61.3)	51,753 (48.3)	124,078 (44.0)	55,557 (51.4)
**Sex**									
Female	145,932 (51.4)	54,087 (60.5)	84,049 (53.1)	32,931 (53.2)		162,664 (49.0)	64,864 (60.6)	148,010 (52.5)	57,301 (53.0)
Male	138,184 (48.6)	35,320 (39.5)	74,256 (46.9)	28,979 (46.8)		169,268 (51.0)	42,190 (39.4)	133,961 (47.5)	50,816 (47.0)
**Insurance**									
Commercial	141,688 (49.9)	60,395 (67.6)	108,336 (68.4)	41,419 (66.9)		128,960 (38.9)	57,973 (54.2)	152,136 (54.0)	55,234 (51.1)
Medicare	129,731 (45.7)	26,673 (29.8)	43,811 (27.7)	17,304 (28.0)		177,618 (53.5)	39,348 (36.8)	90,003 (31.9)	41,125 (38.1)
Medi-Cal	2,068 (0.7)	1,623 (1.8)	2,801 (1.8)	704 (1.1)		11,288 (3.4)	8,169 (7.6)	31,669 (11.2)	6,702 (6.2)
Private pay	10,629 (3.7)	716 (0.8)	3,377 (2.1)	2,483 (4.0)		13,873 (4.2)	1,496 (1.4)	7,973 (2.8)	4,995 (4.6)
Unknown	0 (0.0)	0 (0.0)	0 (0.0)	0 (0.0)		193 (0.1)	68 (0.1)	190 (0.1)	61 (0.1)
**Chronic Conditions**									
Cardiovascular disease	60,874 (21.4)	13,956 (15.6)	21,593 (13.6)	8,230 (13.3)		77,961 (23.5)	19,037 (17.8)	41,929 (14.9)	17,117 (15.8)
Chronic kidney disease	29,822 (10.5)	11,129 (12.4)	16,726 (10.6)	6,908 (11.2)		25,886 (7.8)	10,018 (9.4)	22,723 (8.1)	10,176 (9.4)
Heart failure	16,131 (5.7)	4,931 (5.5)	5,615 (3.5)	1,880 (3.0)		29,574 (8.9)	9,024 (8.4)	15,493 (5.5)	5,376 (5.0)
Diabetes	62,528 (22.0)	23,999 (26.8)	57,903 (36.6)	19,245 (31.1)		94,599 (28.5)	39,687 (37.1)	126,089 (44.7)	46,389 (42.9)
**SBP, mean (SD), mm Hg**	129 (15)	132 (16)	129 (15)	128 (15)		129 (13)	131 (14)	130 (13)	129 (13)
**DBP, mean (SD), mm Hg**	73 (11)	76 (12)	74 (11)	74 (11)		72 (11)	74 (12)	73 (12)	73 (11)
**No. of Medications**									
0	31,001 (10.9)	9,876 (11.0)	22,439 (14.2)	6,203 (10.0)		41,265 (12.4)	13,070 (12.2)	40,281 (14.3)	10,553 (9.8)
1	74,485 (26.2)	19,588 (21.9)	45,913 (29.0)	19,501 (31.5)		99,813 (30.1)	24,614 (23)	91,090 (32.3)	37,747 (34.9)
2	93,896 (33.0)	27,612 (30.9)	50,556 (31.9)	20,349 (32.9)		110,609 (33.3)	35,213 (32.9)	93,098 (33)	36,664 (33.9)
≥ 3	84,734 (29.8)	32,331 (36.2)	39,417 (24.9)	15,857 (25.6)		80,245 (24.2)	34,157 (31.9)	57,501 (20.4)	23,153 (21.4)
Unknown	0 (0.0)	0 (0.0)	0 (0.0)	0 (0.0)		0 (0.0)	0 (0.0)	1 (0.0)	0 (0.0)

Data are expressed as N (%) unless otherwise indicated. NHB = Non-Hispanic Black, NHW = Non-Hispanic White, API = Asian/Pacific Islander
SBP = systolic blood pressure; DBP = diastolic blood pressure

© 2023 Harrison TN et al. *JAMA Network Open.*

eTable 5. Age-Adjusted Proportion of Patients with Blood Pressure Control Among Adults in the Kaiser Permanente Southern California Hypertension Registry, 2008-2019[a]

		2008	2009	2010	2011	2012	2013
**Overall**		74.3 (74.1-74.6)	77.8 (77.6-78.0)	79.7 (79.5-80.0)	81.4 (81.2- 8.6)	81.7 (81.5-81.9)	83.0 (82.8-83.2)
	Female	74.6 (74.3-74.9)	78.1 (77.8-78.4)	79.9 (79.6-80.2)	81.6 (81.3-81.9)	82.0 (81.7-82.3)	83.3 (83.0-83.6)
	Male	74.3 (73.9-74.6)	77.7 (77.3-78.0)	79.7 (79.4-80.0)	81.3 (81.0-81.6)	81.6 (81.2-81.9)	82.9 (82.6-83.2)
**NHB**		69.4 (68.9-70.0)	73.7 (73.1-74.3)	75.6 (75.1-76.2)	77.5 (76.9-78.1)	78.9 (78.3-79.4)	80.0 (79.5-80.6)
	Female	69.9 (69.1-70.6)	74.4 (73.6-75.1)	76.2 (75.4-76.9)	77.9 (77.1-78.6)	79.4 (78.6-80.1)	80.5 (79.7-81.2)
	Male	68.7 (67.8-69.6)	72.7 (71.8-73.6)	74.8 (73.9-75.7)	77.0 (76.1-77.9)	78.1 (77.2-79.0)	79.4 (78.5-80.3)
**NHW**		75.6 (75.2-75.9)	78.9 (78.6-79.3)	80.8 (80.5-81.2)	82.3 (82.0-82.6)	82.2 (81.8-82.5)	83.6 (83.3-84.0)
	Female	75.6 (75.1-76.0)	79.1 (78.6-79.5)	80.7 (80.3-81.2)	82.3 (81.8-82.8)	82.2 (81.7-82.6)	83.7 (83.2-84.1)
	Male	75.8 (75.4-76.3)	79.1 (78.7-79.6)	81.2 (80.8-81.7)	82.6 (82.1-83.0)	82.4 (81.9-82.8)	83.9 (83.4-84.3)
**Hispanic**		74.6 (74.1-75.0)	78.0 (77.6-78.5)	79.7 (79.2-80.1)	81.6 (81.1-82.0)	82.0 (81.5-82.4)	83.1 (82.7-83.5)
	Female	75.6 (74.9-76.2)	79.0 (78.3-79.6)	80.6 (80.0-81.2)	82.5 (81.9-83.1)	82.8 (82.2-83.4)	84.1 (83.5-84.6)
	Male	73.6 (72.9-74.3)	77.1 (76.4-77.8)	78.9 (78.2-79.5)	80.7 (80.0-81.3)	81.2 (80.6-81.8)	82.2 (81.6-82.8)
**API**		77.5 (76.7-78.2)	80.3 (79.6-81.0)	82.5 (81.8-83.2)	83.5 (82.9-84.2)	84.0 (83.3-84.7)	85.3 (84.7-85.9)
	Female	77.5 (76.5-78.5)	80.2 (79.3-81.2)	82.6 (81.7-83.6)	83.4 (82.4-84.3)	84.0 (83.1-84.9)	85.1 (84.2-86.0)
	Male	77.4 (76.4-78.5)	80.4 (79.4-81.5)	82.4 (81.4-83.4)	83.8 (82.8-84.7)	84.1 (83.1-85.0)	85.6 (84.6-86.5)
**Other**		67.8 (66.6-68.9)	70.9 (69.7-72.2)	73.0 (71.6-74.3)	74.0 (72.6-75.5)	75.0 (73.6-76.4)	77.3 (75.9-78.7)
	Female	68.4 (66.7-70.0)	71.7 (69.9-73.5)	73.5 (71.6-75.5)	75.2 (73.1-77.3)	75.9 (73.8-77.9)	78.0 (76.0-80.0)
	Male	67.6 (65.9-69.2)	70.6 (68.8-72.4)	72.8 (70.9-74.7)	73.4 (71.4-75.4)	74.5 (72.6-76.5)	76.8 (74.9-78.8)

[a]Blood pressure control was defined as systolic blood pressure <140 mm Hg and diastolic blood pressure <90 mm Hg. Age-adjustment was conducted using direct standardization using the hypertension registry population from 2017 to 2019.
NHB = Non-Hispanic Black, NHW = Non-Hispanic White, API = Asian/Pacific Islander, Other = Native American/Alaskan Native and multiple or other races and ethnicities

© 2023 Harrison TN et al. *JAMA Network Open.*

eTable 5. Age-Adjusted Proportion of Patients with Blood Pressure Control Among Adults in the Kaiser Permanente Southern California Hypertension Registry, 2008-2019[a] (continued)

		2014	2015	2016	2017	2018	2019
**Overall**		81.1 (80.9-81.3)	78.9 (78.7-79.1)	79.0 (78.8-79.2)	78.4 (78.2-78.5)	79.6 (79.4-79.8)	79.3 (79.1-79.4)
	Female	81.2 (80.9-81.5)	79.0 (78.8-79.3)	79.0 (78.7-79.3)	78.3 (78.0-78.5)	79.7 (79.4-80.0)	79.4 (79.1-79.6)
	Male	81.2 (80.9-81.5)	79.0 (78.7-79.3)	79.3 (79.0-79.6)	78.6 (78.4-78.9)	79.8 (79.5-80.1)	79.4 (79.1 79.6)
**NHB**		78.7 (78.2-79.3)	76.9 (76.4-77.4)	77.0 (76.4-77.5)	76.2 (75.7-76.7)	77.4 (76.9-78.0)	76.6 (76.1-77.2)
	Female	79.0 (78.3-79.7)	77.3 (76.6-78.0)	77.0 (76.3-77.7)	76.2 (75.5-76.9)	77.6 (76.9-78.2)	76.8 (76.1-77.5)
	Male	78.4 (77.5-79.3)	76.4 (75.5-77.3)	77.1 (76.2-77.9)	76.2 (75.4-77.1)	77.4 (76.5-78.2)	76.5 (75.6-77.3)
**NHW**		81.4 (81.1-81.7)	79.1 (78.8-79.4)	79.2 (78.9-79.5)	78.6 (78.3-78.9)	80.1 (79.8-80.4)	79.7 (79.4-80.0)
	Female	81.3 (80.8-81.7)	78.9 (78.5-79.4)	78.8 (78.4-79.3)	78.3 (77.8-78.7)	79.7 (79.3-80.2)	79.5 (79.1-80.0)
	Male	81.9 (81.4-82.3)	79.6 (79.2-80.0)	79.9 (79.5-80.3)	79.3 (78.8-79.7)	80.7 (80.2-81.1)	80.2 (79.7-80.6)
**Hispanic**		81.4 (81.0-81.8)	79.2 (78.9-79.6)	79.4 (79.0-79.7)	78.4 (78.0-78.7)	79.5 (79.2-79.9)	79.3 (79.0-79.7)
	Female	82.1 (81.6-82.7)	80.0 (79.5-80.5)	79.8 (79.3-80.3)	78.8 (78.3-79.3)	80.3 (79.9-80.8)	80.0 (79.6-80.5)
	Male	80.8 (80.2-81.4)	78.6 (78.1-79.2)	79.0 (78.5-79.6)	78.1 (77.6-78.6)	78.9 (78.4-79.4)	78.8 (78.3-79.3)
**API**		83.1 (82.5-83.7)	80.9 (80.3-81.5)	81.0 (80.5-81.6)	80.4 (79.9-81.0)	81.3 (80.8-81.9)	80.8 (80.3-81.4)
	Female	82.8 (81.9-83.6)	80.5 (79.7-81.3)	80.9 (80.1-81.7)	80.1 (79.3-80.8)	80.9 (80.1-81.6)	80.6 (79.8-81.3)
	Male	83.5 (82.6-84.4)	81.4 (80.5-82.3)	81.3 (80.4-82.1)	80.9 (80.1-81.7)	81.9 (81.1-82.7)	81.2 (80.5-82.0)
**Other**		75.9 (74.6-77.3)	74.2 (73.0-75.5)	74.0 (72.9-75.1)	74.5 (73.2-75.8)	76.7 (75.5-77.9)	76.4 (75.3-77.6)
	Female	76.8 (74.9-78.7)	74.5 (72.7-76.2)	74.5 (72.9-76.2)	75.0 (73.2-76.9)	77.1 (75.4-78.8)	76.7 (75.1-78.4)
	Male	75.5 (73.7-77.4)	74.3 (72.6-76.0)	73.8 (72.2-75.4)	74.3 (72.6-76.1)	76.7 (75.0-78.3)	76.4 (74.9-77.9)

[a]Blood pressure control was defined as systolic blood pressure <140 mm Hg and diastolic blood pressure <90 mm Hg. Age-adjustment was conducted using direct standardization using the hypertension registry population from 2017 to 2019.
NHB = Non-Hispanic Black, NHW = Non-Hispanic White, API = Asian/Pacific Islander, Other = Native American/Alaskan Native and multiple or other races and ethnicities

© 2023 Harrison TN et al. *JAMA Network Open.*

eTable 6. Age-Adjusted Proportion of Patients with Blood Pressure Control Among Adults in the Kaiser Permanente Southern California Hypertension Registry and Treated with Antihypertensive Medication, 2008-2019[a]

		2008	2009	2010	2011	2012	2013
**Overall**		75.3 (75.1-75.5)	78.8 (78.6-79.0)	80.6 (80.4-80.9)	82.4 (82.2-82.6)	82.8 (82.6-83.1)	84.3 (84.0-84.5)
	Female	75.2 (74.9-75.5)	78.8 (78.5-79.1)	80.5 (80.2-80.9)	82.3 (82.0-82.7)	82.8 (82.5-83.1)	84.2 (83.9-84.5)
	Male	75.6 (75.2-75.9)	78.9 (78.6-79.3)	80.9 (80.6-81.2)	82.6 (82.3-82.9)	83.0 (82.7-83.3)	84.5 (84.1-84.8)
**NHB**		70.5 (69.9-71.1)	74.7 (74.1-75.4)	76.5 (75.9-77.2)	78.6 (78.0-79.2)	80.0 (79.4-80.6)	81.4 (80.8-82.0)
	Female	70.7 (69.9-71.4)	75.1 (74.3-75.9)	76.9 (76.1-77.6)	78.7 (77.9-79.5)	80.3 (79.5-81.0)	81.5 (80.7-82.3)
	Male	70.1 (69.2-71.1)	74.1 (73.1-75.1)	76.1 (75.1-77.0)	78.4 (77.4-79.4)	79.6 (78.6-80.6)	81.3 (80.3-82.3)
**NHW**		76.3 (76.0-76.7)	79.7 (79.4-80.1)	81.5 (81.2-81.9)	83.1 (82.8-83.5)	83.1 (82.8-83.4)	84.7 (84.4-85.1)
	Female	76.1 (75.6-76.6)	79.6 (79.1-80.1)	81.2 (80.7-81.7)	83.0 (82.5-83.5)	82.9 (82.4-83.4)	84.5 (84.0-85.0)
	Male	76.9 (76.4-77.4)	80.1 (79.6-80.6)	82.1 (81.6-82.6)	83.5 (83.0-84.0)	83.5 (83.0-84.0)	85.2 (84.7-85.6)
**Hispanic**		75.5 (75.0-76.0)	79.0 (78.5-79.5)	80.6 (80.2-81.1)	82.6 (82.2-83.1)	83.1 (82.7-83.6)	84.4 (83.9-84.8)
	Female	76.0 (75.4-76.7)	79.5 (78.8-80.1)	81.1 (80.5-81.8)	83.2 (82.5-83.8)	83.6 (83.0-84.2)	84.9 (84.3-85.5)
	Male	74.9 (74.2-75.7)	78.5 (77.8-79.2)	80.2 (79.5-80.9)	82.2 (81.5-82.9)	82.8 (82.1-83.5)	83.9 (83.3-84.6)
**API**		78.5 (77.8-79.3)	81.3 (80.6-82.1)	83.4 (82.7-84.2)	84.5 (83.8-85.2)	85.1 (84.4-85.8)	86.4 (85.7-87.1)
	Female	78.4 (77.3-79.4)	81.1 (80.0-82.1)	83.4 (82.4-84.4)	84.2 (83.2-85.2)	84.9 (84.0-85.9)	86.0 (85.1-86.9)
	Male	78.7 (77.5-79.8)	81.6 (80.5-82.7)	83.5 (82.4-84.5)	84.9 (83.8-85.9)	85.3 (84.2-86.3)	86.9 (85.9-87.9)
**Other**		71.2 (69.9-72.5)	75.1 (73.7-76.5)	76.6 (75.1-78.1)	78.3 (76.7-79.9)	79.5 (78.0-81.1)	81.3 (79.8-82.9)
	Female	71.4 (69.6-73.2)	75.6 (73.6-77.6)	77.0 (74.9-79.2)	78.9 (76.6-81.3)	79.5 (77.2-81.8)	81.6 (79.4-83.9)
	Male	71.2 (69.4-73.1)	75.0 (73.0-77.0)	76.5 (74.4-78.6)	78.1 (75.8-80.4)	79.8 (77.6-82.0)	81.2 (79.1-83.3)

[a]Blood pressure control was defined as systolic blood pressure <140 mm Hg and diastolic blood pressure <90 mm Hg. Age-adjusted rate was obtained by applying direct standardization using combined HTN registry population from 2017 to 2019.
NHB = Non-Hispanic Black, NHW = Non-Hispanic White, API = Asian/Pacific Islander, Other = Native American/Alaskan Native and multiple or other races and ethnicities

© 2023 Harrison TN et al. *JAMA Network Open.*

eTable 6. Age-Adjusted Proportion of Patients with Blood Pressure Control Among Adults in the Kaiser Permanente Southern California Hypertension Registry and Treated with Antihypertensive Medication, 2008-2019[a] (continued)

		2014	2015	2016	2017	2018	2019
**Overall**		82.5 (82.2-82.7)	80.5 (80.3-80.7)	80.6 (80.4-80.8)	80.2 (80.0-80.4)	81.3 (81.1-81.5)	80.9 (80.7-81.1)
	Female	82.2 (81.9-82.5)	80.2 (80.0-80.5)	80.2 (79.9-80.5)	79.7 (79.5-80.0)	81.0 (80.7-81.2)	80.7 (80.4-80.9)
	Male	82.9 (82.6-83.2)	81.0 (80.7-81.3)	81.2 (80.9-81.6)	80.8 (80.5-81.1)	81.9 (81.6-82.2)	81.4 (81.1-81.7)
**NHB**		80.2 (79.6-80.8)	78.5 (77.9-79.1)	78.5 (78.0-79.1)	78.1 (77.5-78.7)	79.2 (78.6-79.7)	78.4 (77.8-79.0)
	Female	80.1 (79.4-80.9)	78.5 (77.8-79.3)	78.2 (77.5-78.9)	77.8 (77.1-78.5)	78.9 (78.2-79.6)	78.2 (77.4-78.9)
	Male	80.3 (79.4-81.3)	78.4 (77.5-79.4)	79.2 (78.3-80.1)	78.6 (77.7-79.6)	79.7 (78.8-80.7)	78.9 (78.0-79.8)
**NHW**		82.6 (82.2-82.9)	80.5 (80.1-80.8)	80.6 (80.3-81.0)	80.2 (79.9-80.6)	81.6 (81.2-81.9)	81.2 (80.9-81.5)
	Female	82.1 (81.6-82.6)	80.0 (79.5-80.5)	80.0 (79.5-80.5)	79.6 (79.1-80.1)	80.9 (80.4-81.4)	80.7 (80.2-81.2)
	Male	83.3 (82.8-83.7)	81.2 (80.8-81.7)	81.6 (81.1-82.1)	81.1 (80.6-81.6)	82.4 (82.0-82.9)	81.9 (81.5-82.4)
**Hispanic**		82.8 (82.4-83.2)	80.9 (80.5-81.3)	80.9 (80.5-81.3)	80.3 (79.9-80.7)	81.3 (81.0-81.7)	81.1 (80.7-81.4)
	Female	83.1 (82.5-83.7)	81.2 (80.6-81.7)	80.9 (80.4-81.5)	80.2 (79.7-80.8)	81.5 (81.0-82.1)	81.3 (80.8-81.8)
	Male	82.7 (82.1-83.3)	80.8 (80.2-81.4)	81.1 (80.6-81.7)	80.6 (80.0-81.1)	81.3 (80.7-81.8)	81.1 (80.6-81.6)
**API**		84.4 (83.7-85.0)	82.5 (81.8-83.1)	82.6 (82.0-83.2)	82.2 (81.6-82.8)	82.9 (82.3-83.4)	82.5 (81.9-83.0)
	Female	84.0 (83.1-84.9)	81.9 (81.0-82.7)	82.3 (81.4-83.1)	81.7 (80.8-82.5)	82.2 (81.4-83.0)	82.1 (81.3-82.9)
	Male	84.9 (83.9-85.9)	83.2 (82.3-84.1)	83.0 (82.1-83.9)	82.8 (82.0-83.7)	83.7 (82.9-84.6)	83.0 (82.2-83.8)
**Other**		80.1 (78.7-81.6)	78.0 (76.7-79.4)	77.5 (76.2-78.7)	78.0 (76.6-79.4)	79.8 (78.5-81.1)	79.6 (78.4-80.8)
	Female	80.6 (78.5-82.7)	77.6 (75.7-79.6)	77.5 (75.7-79.3)	77.9 (75.9-80.0)	79.6 (77.7-81.5)	79.6 (77.9-81.4)
	Male	79.9 (77.9-81.9)	78.6 (76.7-80.5)	77.6 (75.9-79.4)	78.3 (76.4-80.2)	80.2 (78.4-82.0)	79.7 (78.0-81.4)

[a] Blood pressure control was defined as systolic blood pressure <140 mm Hg and diastolic blood pressure <90 mm Hg. Age-adjusted rate was obtained by applying direct standardization using combined HTN registry population from 2017 to 2019.
NHB = Non-Hispanic Black, NHW = Non-Hispanic White, API = Asian/Pacific Islander, Other = Native American/Alaskan Native and multiple or other races and ethnicities

© 2023 Harrison TN et al. *JAMA Network Open.*

eFigure 1. Age-Adjusted Proportion of Patients with Blood Pressure Control by Sex and Race and Ethnicity Among Adults in the Kaiser Permanente Southern California Hypertension Registry and Treated with Antihypertensive Medication, 2008-2019

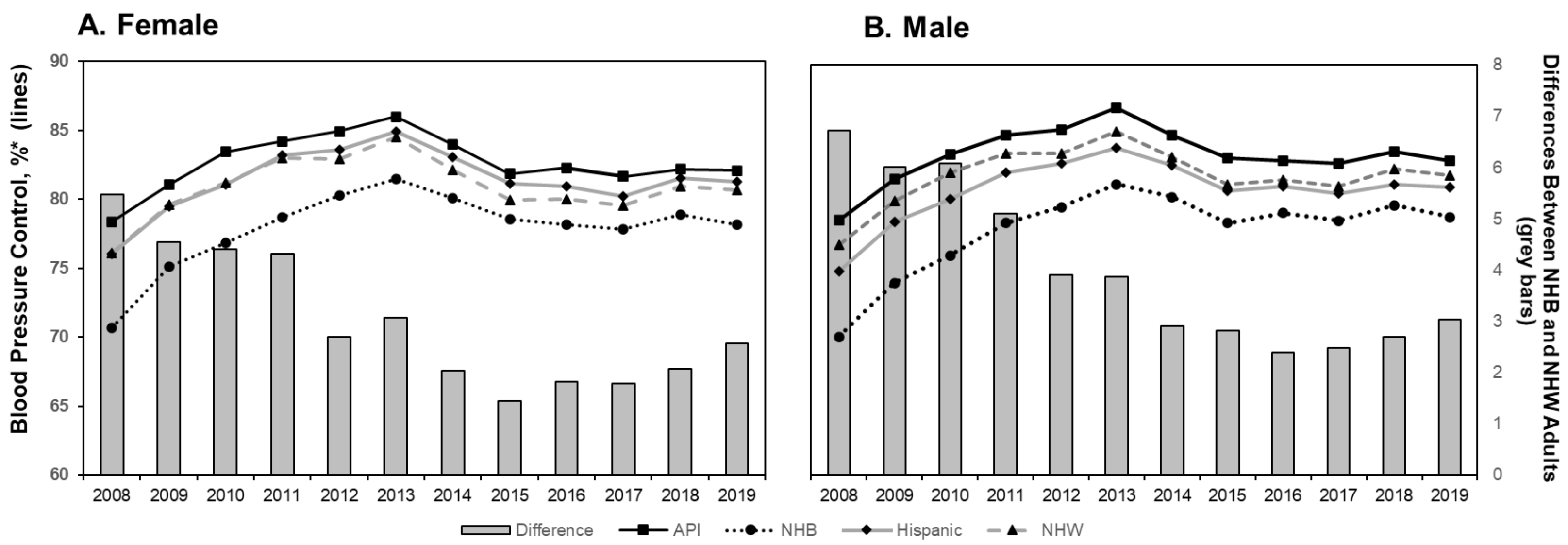

NHB = Non-Hispanic Black, NHW = Non-Hispanic White, API = Asian/Pacific Islander

© 2023 Harrison TN et al. *JAMA Network Open.*

eTable 7. Age-Specific Proportion of Patients with Blood Pressure Control by Sex and Race and Ethnicity Among Adults in the Kaiser Permanente Southern California Hypertension Registry, 2008-2019[a]

	2008	2009	2010	2011	2012	2013
**Overall**						
Male						
18-49	65.9 (65.5-66.3)	68.7 (68.4-69.1)	70.5 (70.1-70.8)	71.7 (71.3-72.1)	71.7 (71.3-72.1)	72.5 (72.1-72.9)
50-64	72.2 (71.9-72.4)	75.5 (75.3-75.8)	77.6 (77.4-77.8)	79.0 (78.8-79.2)	79.1 (78.9-79.3)	80.4 (80.2-80.6)
65+	78.0 (77.7-78.2)	81.6 (81.4-81.8)	83.7 (83.5-83.9)	85.5 (85.4-85.7)	85.9 (85.8-86.1)	87.4 (87.3-87.6)
Female						
18-49	73.1 (72.8-73.5)	75.7 (75.3-76.0)	76.8 (76.4-77.1)	77.5 (77.1-77.8)	77.8 (77.4-78.1)	77.7 (77.3-78.0)
50-64	75.7 (75.4-75.9)	79.1 (78.9-79.3)	80.7 (80.5-80.9)	82.0 (81.8-82.2)	82.0 (81.8-82.2)	83.1 (82.9-83.3)
65+	74.2 (74.0-74.5)	78.2 (78.0-78.4)	80.2 (80.0-80.4)	82.4 (82.2-82.6)	83.1 (82.9-83.3)	85.0 (84.8-85.2)
**NHB**						
Male						
18-49	58.3 (57.2-59.4)	62.0 (60.9-63.1)	64.5 (63.4-65.5)	65.1 (64.0-66.2)	66.7 (65.6-67.8)	66.2 (65.0-67.3)
50-64	66.7 (65.9-67.5)	70.1 (69.4-70.8)	72.1 (71.3-72.8)	73.8 (73.1-74.5)	75.3 (74.6-75.9)	76.5 (75.8-77.1)
65+	72.9 (72.2-73.7)	77.4 (76.7-78.1)	79.5 (78.9-80.2)	82.4 (81.8-83.0)	83.1 (82.6-83.7)	85.1 (84.5-85.6)
Female						
18-49	68.3 (67.5-69.2)	70.3 (69.5-71.1)	71.7 (70.8-72.5)	73.2 (72.4-74.0)	74.5 (73.6-75.3)	74.7 (73.8-75.5)
50-64	70.2 (69.6-70.8)	74.3 (73.7-74.9)	75.9 (75.3-76.4)	77.6 (77.1-78.1)	78.2 (77.7-78.8)	79.3 (78.8-79.8)
65+	70.1 (69.5-70.8)	75.6 (75.0-76.1)	77.6 (77.1-78.2)	79.4 (78.9-79.9)	81.5 (81.0-82.0)	82.9 (82.4-83.3)
**NHW**						
Male						
18-49	68.0 (67.3-68.7)	70.7 (70.0-71.4)	72.3 (71.7-73.0)	73.3 (72.6-73.9)	73.0 (72.3-73.6)	74.0 (73.4-74.7)
50-64	73.4 (73.1-73.8)	77.0 (76.6-77.3)	79.4 (79.0-79.7)	80.2 (79.9-80.6)	79.6 (79.3-79.9)	81.4 (81.0-81.7)
65+	79.6 (79.3-79.9)	82.9 (82.6-83.2)	85.0 (84.7-85.2)	86.7 (86.5-87.0)	86.9 (86.7-87.1)	88.2 (88.0-88.5)
Female						
18-49	74.7 (74.0-75.4)	78.2 (77.5-78.9)	79.1 (78.4-79.7)	78.7 (78.1-79.4)	78.8 (78.1-79.4)	78.4 (77.7-79.1)
50-64	76.9 (76.6-77.3)	80.0 (79.6-80.3)	81.7 (81.3-82.0)	83.0 (82.7-83.3)	82.1 (81.8-82.4)	83.6 (83.3-83.9)
65+	74.9 (74.6-75.2)	78.7 (78.4-79.0)	80.6 (80.3-80.8)	82.8 (82.6-83.1)	83.2 (83.0-83.5)	85.2 (84.9-85.4)
**Hispanic**						
Male						
18-49	66.9 (66.3-67.6)	70.0 (69.4-70.7)	70.9 (70.3-71.5)	72.5 (71.9-73.1)	71.8 (71.2-72.4)	72.2 (71.6-72.8)
50-64	72.2 (71.8-72.7)	75.3 (74.8-75.8)	76.8 (76.3-77.2)	78.7 (78.3-79.1)	79.3 (78.9-79.7)	79.8 (79.4-80.2)
65+	76.3 (75.8-76.9)	80.3 (79.8-80.8)	82.5 (82.1-83.0)	84.3 (83.9-84.7)	85.1 (84.7-85.4)	86.6 (86.2-86.9)

© 2023 Harrison TN et al. *JAMA Network Open.*

Female						
18-49	75.4 (74.8-76.0)	77.5 (76.9-78.1)	78.3 (77.8-78.9)	78.8 (78.2-79.4)	78.9 (78.4-79.5)	78.9 (78.3-79.5)
50-64	77.1 (76.6-77.5)	80.3 (79.9-80.7)	81.8 (81.4-82.2)	83.2 (82.8-83.6)	83.5 (83.1-83.8)	83.9 (83.6-84.3)
65+	74.6 (74.1-75.1)	78.4 (78.0-78.9)	80.4 (79.9-80.8)	83.0 (82.6-83.4)	83.5 (83.1-83.8)	85.6(85.3-85.9)
**API**						
Male						
18-49	71.2 (70.0-72.4)	72.6 (71.4-73.7)	74.4 (73.3-75.5)	76.0 (75.0-77.1)	75.9 (74.9-76.9)	77.5 (76.5-78.5)
50-64	76.4 (75.7-77.2)	79.5 (78.9-80.2)	81.1 (80.5-81.8)	83.0 (82.3-83.6)	83.1 (82.4-83.7)	84.1 (83.5-84.7)
65+	79.8 (79.1-80.6)	83.2 (82.5-83.9)	85.4 (84.8-86.0)	86.4 (85.9-87.0)	87.0 (86.5-87.5)	88.8 (88.4-89.3)
Female						
18-49	75.5(74.3-76.6)	77.1 (76.0-78.2)	78.9 (77.8-79.9)	79.2 (78.2-80.2)	79.0 (78.0-80.0)	78.7 (77.6-79.7)
50-64	78.9(78.3-79.6)	82.1 (81.6-82.7)	83.6 (83.0-84.1)	84.2 (83.6-84.7)	84.5 (83.9-85.0)	85.6 (85.1-86.1)
65+	77.1(76.4-77.9)	79.8 (79.2-80.5)	83.0 (82.4-83.6)	84.0 (83.4-84.6)	85.0 (84.5-85.6)	86.5 (86.0-87.0)
**Other**						
Male						
18-49	60.5 (59.2-61.8)	62.1 (60.7-63.4)	65.3(63.8-66.8)	63.5 (61.8-65.3)	65.3 (63.6-67.1)	68.7 (67.0-70.5)
50-64	66.2 (65.2-67.3)	68.9 (67.8-70.0)	71.4 (70.3-72.6)	71.2 (69.9-72.5)	72.0 (70.7-73.2)	74.7 (73.5-76.0)
65+	70.4 (68.8-72.0)	74.1 (72.5-75.7)	75.7 (74.1-77.4)	77.7 (76.0-79.3)	78.8 (77.3-80.3)	80.5 (79.1-81.9)
Female						
18-49	66.5 (64.8-68.1)	69.2 (67.5-70.9)	69.1 (67.2-71.0)	73.5 (71.5-75.6)	73.0 (70.8-75.1)	72.3 (70.1-74.5)
50-64	68.9 (67.8-70.0)	72.9 (71.8-74.1)	74.7 (73.4-75.9)	74.6 (73.2-76.0)	76.8 (75.5-78.2)	78.5 (77.2-79.9)
65+	68.5 (67.0-70.1)	71.5 (69.9-73.2)	73.9 (72.3-75.6)	76.1 (74.4-77.8)	76.1 (74.4-77.7)	79.3(77.8-80.7)

[a]Blood pressure control was defined as systolic blood pressure <140 mm Hg and diastolic blood pressure <90 mm Hg.
NHB = Non-Hispanic Black, NHW = Non-Hispanic White, API = Asian/Pacific Islander, Other = Native American/Alaskan Native and multiple or other races and ethnicities

© 2023 Harrison TN et al. *JAMA Network Open.*

eTable 7. Age-Specific Proportion of Patients with Blood Pressure Control by Sex and Race/Ethnicity Among Adults in the Kaiser Permanente Southern California Hypertension Registry, 2008-2019[a] (continued)

	2014	2015	2016	2017	2018	2019
**Overall**						
Male						
18-49	70.4 (70.1-70.8)	68.0 (67.6-68.4)	68.0(67.7-68.4)	67.2 (66.8-67.6)	67.8 (67.5-68.2)	67.1 (66.7-67.4)
50-64	78.9 (78.7-79.1)	76.5 (76.2-76.7)	76.5 (76.3-76.7)	76.1 (75.9-76.3)	76.8 (76.6-77.0)	75.9 (75.7-76.1)
65+	85.7 (85.5-85.9)	83.8 (83.6-83.9)	84.3 (84.1-84.4)	83.5 (83.4-83.7)	85.1 (84.9-85.3)	85.1 (84.9-85.2)
Female						
18-49	76.2 (75.8-76.5)	74.0 (73.7-74.4)	73.2 (72.9-73.6)	72.1 (71.7-72.5)	72.9 (72.6-73.3)	71.9 (71.6-72.3)
50-64	81.6 (81.3-81.8)	79.5 (79.3-79.7)	79.6 (79.4-79.8)	78.6 (78.4-78.8)	79.5 (79.3-79.7)	79.0 (78.8-79.2)
65+	82.4 (82.2-82.5)	80.2 (80.0-80.3)	80.2 (80.0-80.3)	79.8 (79.6-80.0)	81.7 (81.5-81.8)	81.6 (81.5-81.8)
**NHB**						
Male						
18-49	65.4 (64.2-66.5)	64.9 (63.7-66.0)	64.5 (63.3-65.6)	64.0 (62.8-65.2)	63.4 (62.2-64.6)	63.0 (61.8-64.2)
50-64	76.1 (75.4-76.8)	73.8 (73.1-74.5)	74.2 (73.5-74.9)	73.6 (72.9-74.3)	74.4 (73.7-75.0)	72.1 (71.4-72.8)
65+	83.5 (83.0-84.1)	81.4 (80.8-82.0)	82.5 (81.9-83.0)	81.4 (80.8-81.9)	83.2 (82.7-83.8)	83.1 (82.6-83.6)
Female						
18-49	73.3 (72.5-74.2)	71.9 (71.0-72.8)	71.4 (70.5-72.3)	69.5 (68.5-70.4)	70.3 (69.4-71.2)	67.9 (66.9-68.9)
50-64	78.5 (77.9-79.0)	77.2 (76.7-77.7)	77.2 (76.7-77.7)	76.0 (75.4-76.5)	76.8 (76.3-77.3)	75.9 (75.4-76.5)
65+	80.9 (80.4-81.4)	78.8 (78.3-79.3)	78.4 (77.9-78.9)	78.3 (77.8-78.7)	80.1 (79.7-80.5)	79.9 (79.4-80.3)
**NHW**						
Male						
18-49	71.6 (70.9-72.3)	69.1 (68.4-69.8)	69.2 (68.5-69.9)	68.4 (67.7-69.0)	69.5 (68.8-70.2)	68.1 (67.4-68.8)
50-64	79.5 (79.1-79.8)	76.8 (76.5-77.1)	76.8 (76.5-77.2)	76.5 (76.2-76.9)	77.5 (77.1-77.8)	76.6 (76.3-77.0)
65+	86.3 (86.1-86.6)	84.4 (84.2-84.6)	84.9 (84.7-85.2)	84.1 (83.9-84.3)	85.9 (85.7-86.1)	85.9 (85.7-86.1)
Female						
18-49	76.8 (76.1-77.5)	74.8 (74.1-75.6)	73.8 (73.0-74.6)	72.7 (71.9-73.5)	73.9 (73.1-74.6)	72.9 (72.2-73.7)
50-64	81.7 (81.4-82.1)	79.0 (78.7-79.4)	79.3 (79.0-79.7)	78.4 (78.0-78.7)	79.1 (78.7-79.5)	78.9 (78.5-79.3)
65+	82.2 (82.0-82.5)	80.0 (79.8-80.2)	79.9 (79.7-80.2)	79.8 (79.5-80.0)	81.8 (81.6-82.0)	81.8 (81.5-82.0)
**Hispanic**						
Male						
18-49	70.8 (70.2-71.3)	67.4 (66.8-68.0)	67.8 (67.2-68.3)	66.4 (65.9-67.0)	66.6 (66.0-67.1)	66.6 (66.1-67.2)
50-64	78.6 (78.2-79.0)	76.3 (75.9-76.7)	76.5 (76.2-76.9)	75.7 (75.3-76.1)	76.2 (75.8-76.6)	75.5 (75.1-75.9)
65+	85.0 (84.7-85.4)	83.2 (82.9-83.6)	83.9 (83.5-84.2)	83.0 (82.6-83.3)	84.1 (83.8-84.5)	84.4 (84.1-84.7)

© 2023 Harrison TN et al. *JAMA Network Open.*

Female						
18-49	77.1 (76.5-77.7)	75.0 (74.4-75.6)	73.6 (73.1-74.2)	72.7 (72.1-73.2)	73.5 (73.0-74.1)	72.8 (72.3-73.4)
50-64	82.8 (82.4-83.1)	80.8 (80.4-81.1)	80.6 (80.3-81.0)	79.4 (79.1-79.8)	80.4 (80.1-80.8)	79.9 (79.6-80.3)
65+	83.1 (82.7-83.4)	80.9 (80.5-81.2)	81.0 (80.7-81.3)	80.1 (79.8-80.4)	82.1 (81.8-82.4)	82.1 (81.8-82.4)
**API**						
Male						
18-49	74.0 (72.9-75.0)	72.0 (70.9-73.0)	71.3 (70.3-72.3)	70.8 (69.8-71.8)	72.1 (71.1-73.0)	70.0 (69.0-71.0)
50-64	82.3 (81.7-82.9)	80.1 (79.5-80.7)	79.4 (78.8-80.1)	79.3 (78.7-79.9)	79.7 (79.1-80.3)	79.2 (78.6-79.8)
65+	86.9 (86.4-87.4)	84.9 (84.4-85.4)	85.2 (84.8-85.7)	84.8 (84.3-85.3)	86.1 (85.7-86.6)	85.7 (85.3-86.2)
Female						
18-49	77.3 (76.2-78.3)	74.2 (73.1-75.2)	74.8 (73.8-75.8)	73.1 (72.1-74.2)	73.1 (72.1-74.1)	72.8 (71.9-73.8)
50-64	83.2 (82.6-83.7)	81.5 (80.9-82.1)	81.6 (81.1-82.2)	81.0 (80.4-81.5)	81.8 (81.2-82.3)	80.9 (80.4-81.5)
65+	84.0 (83.5-84.5)	81.5 (81.0-82.0)	82.2 (81.7-82.7)	81.4 (80.9-81.8)	82.4 (81.9-82.8)	82.5 (82.0-82.9)
**Other**						
Male						
18-49	64.2 (62.4-66.0)	63.7 (62.0-65.5)	63.7 (62.0-65.3)	64.2 (62.4-66.0)	67.7 (66.1-69.3)	65.5 (64.0-67.1)
50-64	73.2 (71.9-74.4)	71.5 (70.3-72.7)	71.8 (70.7-73.0)	71.6 (70.3-72.8)	73.9 (72.7-75.0)	74.1 (73.0-75.2)
65+	80.3 (79.0-81.6)	79.2 (77.9-80.4)	77.9 (76.7-79.1)	79.0 (77.7-80.3)	81.0 (79.9-82.2)	81.0 (79.9-82.1)
Female						
18-49	72.9 (70.8-75.1)	68.6 (66.5-70.6)	68.9 (66.9-70.8)	69.6 (67.4-71.9)	71.9 (69.9-74.0)	69.7 (67.8-71.6)
50-64	76.4 (75.1-77.8)	74.6 (73.3-76.0)	75.5 (74.2-76.8)	74.5 (73.0-75.9)	76.6 (75.3-77.9)	76.4 (75.2-77.7)
65+	78.0 (76.6-79.4)	76.0 (74.6-77.3)	75.5 (74.2-76.8)	76.9 (75.5-78.2)	78.8 (77.6-80.1)	78.9 (77.7-80.0)

[a]Blood pressure control was defined as systolic blood pressure <140 mm Hg and diastolic blood pressure <90 mm Hg.
NHB = Non-Hispanic Black, NHW = Non-Hispanic White, API = Asian/Pacific Islander, Other = Native American/Alaskan Native and multiple or other races and ethnicities

© 2023 Harrison TN et al. *JAMA Network Open.*

eFigure 2. **Age-Specific Proportion of Patients with Blood Pressure Control by Sex and Race and Ethnicity Among Adults in the Kaiser Permanente Southern California Hypertension Registry, 2008-2019**

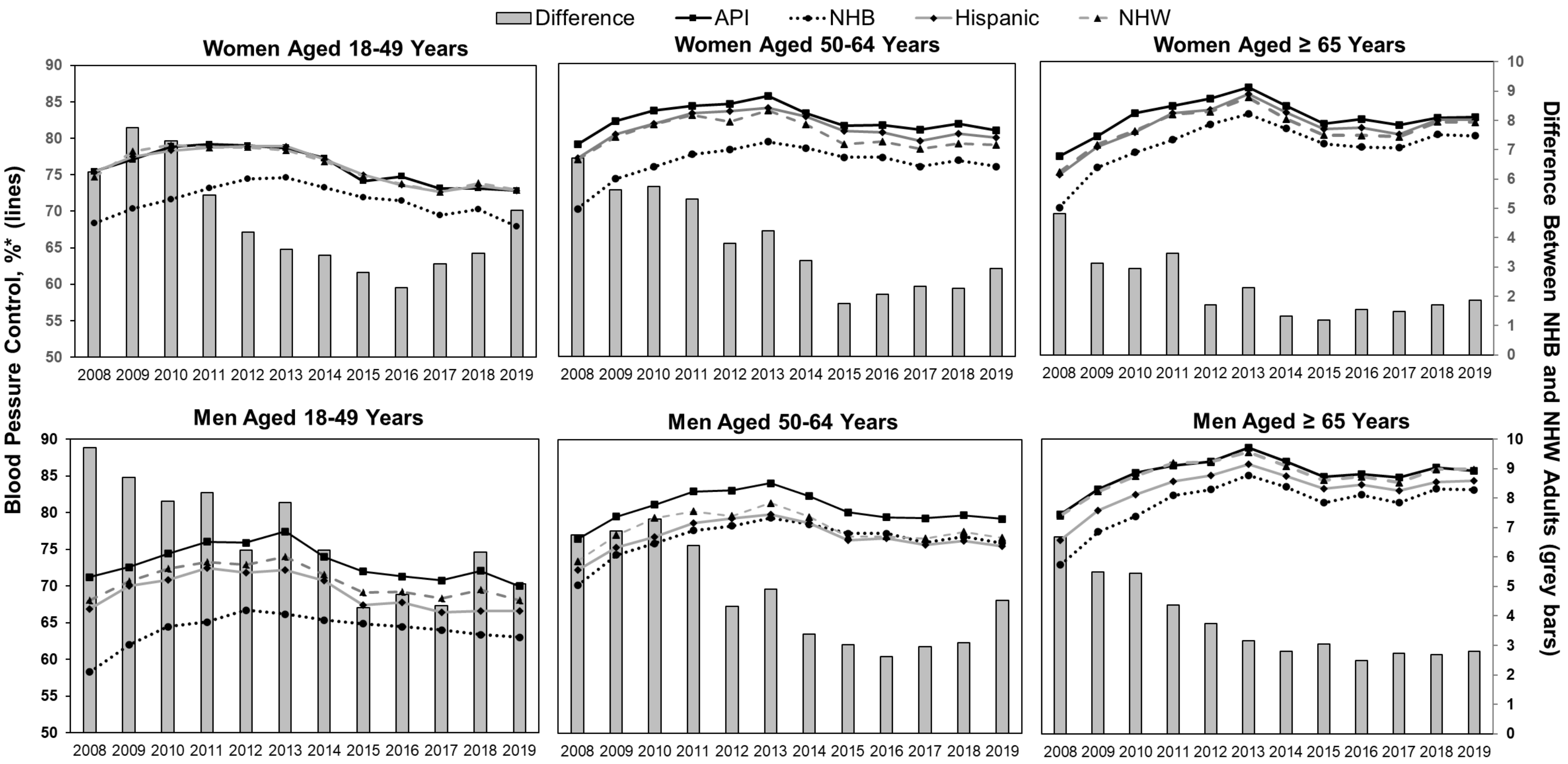

NHB = Non-Hispanic Black, NHW = Non-Hispanic White, API = Asian/Pacific Islander

© 2023 Harrison TN et al. *JAMA Network Open.*

eTable 8. Age-adjusted Proportion of Patients with Blood Pressure Control (<130/80 mm Hg) by Race and Ethnicity Among Adults in the Kaiser Permanente Southern California Hypertension Registry, 2008-2019

	2008	2009	2010	2011	2012	2013
**NHB**	36.8 (36.4-37.2)	40.8 (40.3-41.2)	42.3 (41.9-42.7)	39.7 (39.3-40.1)	38.5 (38.1-38.9)	38.1 (37.7-38.5)
**NHW**	42.7 (42.5-43.0)	46.3 (46.0-46.5)	48.0 (47.7-48.2)	45.2 (44.9-45.4)	43.5 (43.2-43.7)	43.1 (42.9-43.3)
**Hispanic**	43.0 (42.7-43.4)	47.4 (47.0-47.8)	48.9 (48.5-49.2)	45.4 (45.1-45.7)	42.6 (42.3-42.9)	42.2 (41.9-42.5)
**API**	46.2 (45.7-46.8)	49.5 (48.9-50.0)	51.1 (50.5-51.6)	48.0 (47.5-48.5)	45.4 (44.9-45.9)	44.5 (44.1-45.0)

	2014	2015	2016	2017	2018	2019
**NHB**	36.4 (36.1-36.8)	34.7 (34.3-35.1)	34.4 (34.1-34.8)	34.0 (33.6-34.3)	34.3 (34.0-34.7)	33.8 (33.5-34.2)
**NHW**	41.2 (41.0-41.4)	39.0 (38.8-39.2)	38.9 (38.7-39.1)	38.3 (38.1-38.6)	38.7 (38.5-39.0)	38.3 (38.0-38.5)
**Hispanic**	40.4 (40.1-40.7)	38.0 (37.8-38.3)	37.8 (37.5-38.0)	36.9 (36.7-37.1)	36.9 (36.6-37.1)	36.7 (36.5-36.9)
**API**	42.2 (41.7-42.6)	39.5 (39.1-39.9)	39.4 (39.0-39.8)	38.6 (38.2-39.0)	38.4 (38.1-38.8)	37.9 (37.5-38.2)

NHB = Non-Hispanic Black, NHW = Non-Hispanic White, API = Asian/Pacific Islander

© 2023 Harrison TN et al. *JAMA Network Open.*

eTable 9. Factors Associated with Uncontrolled Blood Pressure by Race and Ethnicity Among Adults in the Kaiser Permanente Southern California Hypertension Registry in 2019

	Prevalence Ratio (95% CI)[a]				
	**NHB**	**NHW**	**Hispanic**	**API**	**Other**
**Age group, years**					
18-49	Reference	Reference	Reference	Reference	Reference
50-64	0.82 (0.79-0.84)	0.82 (0.80-0.84)	0.80 (0.79-0.82)	0.74 (0.72-0.77)	0.82 (0.78-0.86)
65+	0.73 (0.71-0.76)	0.76 (0.74-0.78)	0.75 (0.73-0.77)	0.71 (0.68-0.74)	0.82 (0.76-0.89)
**Sex**					
Male	Reference	Reference	Reference	Reference	Reference
Female	1.06 (1.04-1.09)	1.13 (1.11-1.14)	0.99 (0.98-1.01)	1.08 (1.06-1.11)	1.01 (0.97-1.05)
**Chronic Conditions**					
Cardiovascular disease	1.00 (0.97-1.03)	0.99 (0.97-1.00)	0.98 (0.95-1.00)	0.98 (0.94-1.02)	0.97 (0.89-1.05)
Chronic kidney disease	1.27 (1.22-1.31)	1.20 (1.17-1.23)	1.33 (1.29-1.36)	1.31 (1.26-1.37)	1.28 (1.17-1.41)
Heart failure	1.04 (1.00-1.09)	0.98 (0.95-1.01)	0.96 (0.92-1.00)	0.99 (0.92-1.05)	0.95 (0.82-1.10)
Diabetes	1.00 (0.98-1.02)	0.97 (0.95-0.98)	0.94 (0.92-0.95)	0.96 (0.94-0.99)	0.92 (0.88-0.97)
**Insurance**					
Commercial	Reference	Reference	Reference	Reference	Reference
Medicare	0.81 (0.78-0.84)	0.84 (0.82-0.86)	0.84 (0.82-0.87)	0.88 (0.85-0.92)	0.79 (0.73-0.86)
Medi-Cal	0.96 (0.92-1.00)	0.94 (0.90-0.97)	0.90 (0.87-0.92)	0.92 (0.87-0.98)	0.80 (0.71-0.91)
Private pay	0.86 (0.79-0.93)	0.98 (0.96-1.02)	0.90 (0.86-0.94)	0.91 (0.86-0.96)	0.92 (0.84-1.01)
**Number of outpatient visits[b]**					
0	Reference	Reference	Reference	Reference	Reference
1-6	0.69 (0.67-0.71)	0.71 (0.70-0.73)	0.76 (0.75-0.77)	0.74 (0.71-0.76)	0.85 (0.81-0.89)
7-12	0.50 (0.48-0.51)	0.51 (0.49-0.52)	0.55 (0.53-0.56)	0.52 (0.50-0.55)	0.61 (0.57-0.66)
≥13	0.45 (0.43-0.47)	0.43 (0.42-0.44)	0.49 (0.47-0.50)	0.47 (0.45-0.49)	0.55 (0.51-0.60)
**Number of inpatient visits[b]**					
0	Reference	Reference	Reference	Reference	Reference
1-3	1.02 (0.98-1.05)	1.04 (1.02-1.07)	1.00 (0.98-1.02)	0.98 (0.94-1.03)	1.01 (0.93-1.10)
≥3	0.93 (0.85-1.03)	0.91 (0.84-0.99)	0.90 (0.84-0.96)	0.96 (0.84-1.10)	0.73 (0.49-1.08)
**Number of antihypertensive medications**					
0	Reference	Reference	Reference	Reference	Reference
1	0.64 (0.62-0.66)	0.64 (0.63-0.65)	0.61 (0.60-0.62)	0.53 (0.51-0.55)	0.49 (0.47-0.52)
2	0.63 (0.61-0.65)	0.63 (0.62-0.65)	0.63 (0.62-0.64)	0.54 (0.52-0.56)	0.48 (0.46-0.51)
≥3	0.71 (0.69-0.73)	0.70 (0.69-0.72)	0.73 (0.71-0.74)	0.59 (0.57-0.62)	0.54 (0.50-0.57)

NHB = Non-Hispanic Black, NHW = Non-Hispanic White, API = Asian/Pacific Islander, Other = Native American/Alaskan Native and multiple or other races and ethnicities
[a]Adjusted for all variables in the table
[b]Visits in 2018

© 2023 Harrison TN et al. *JAMA Network Open.*

eTable 10. Factors Associated with Uncontrolled Blood Pressure by Race and Ethnicity Among Adults in the Kaiser Permanente Southern California Hypertension Registry and Treated with Antihypertensive Medication in 2019

	Prevalence Ratio (95% CI)[a]				
	**NHB**	**NHW**	**Hispanic**	**API**	**Other**
**Age group, years**					
18-49	Reference	Reference	Reference	Reference	Reference
50-64	0.75 (0.73-0.77)	0.78 (0.76-0.80)	0.76 (0.74-0.77)	0.71 (0.69-0.74)	0.77 (0.73-0.82)
65+	0.65 (0.62-0.68)	0.70 (0.68-0.72)	0.68 (0.66-0.70)	0.66 (0.62-0.69)	0.72 (0.66-0.80)
**Sex**					
Male	Reference	Reference	Reference	Reference	Reference
Female	1.09 (1.06-1.12)	1.16 (1.14-1.18)	1.03 (1.01-1.05)	1.10 (1.07-1.13)	1.01 (0.96-1.06)
**Chronic Conditions**					
Cardiovascular disease	1.00 (0.96-1.03)	1.00 (0.98-1.02)	0.98 (0.96-1.01)	0.98 (0.94-1.02)	0.96 (0.87-1.06)
Chronic kidney disease	1.27 (1.22-1.32)	1.22 (1.18-1.25)	1.34 (1.31-1.38)	1.32 (1.26-1.38)	1.27 (1.14-1.42)
Heart failure	1.05 (1.00-1.10)	0.97(0.94-1.00)	0.94 (0.90-0.98)	0.99 (0.92-1.06)	0.92 (0.78-1.08)
Diabetes	1.02 (0.99-1.04)	0.97 (0.95-0.99)	0.93 (0.92-0.95)	0.96 (0.94-0.99)	0.94 (0.89-1.00)
**Insurance**					
Commercial	Reference	Reference	Reference	Reference	Reference
Medicare	0.83 (0.80-0.86)	0.85 (0.83-0.87)	0.85 (0.82-0.87)	0.88 (0.84-0.93)	0.79 (0.72-0.88)
Medi-Cal	0.98 (0.94-1.03)	0.93 (0.89-0.97)	0.90 (0.87-0.92)	0.94 (0.88-1.00)	0.81 (0.70-0.94)
Private pay	0.85 (0.76-0.93)	0.99 (0.96-1.03)	0.91 (0.87-0.96)	0.91 (0.86-0.97)	0.88 (0.79-0.99)
**Number of outpatient visits[b]**					
0	Reference	Reference	Reference	Reference	Reference
1-6	0.79 (0.76-0.83)	0.85 (0.83-0.88)	0.90 (0.88-0.93)	0.90 (0.86-0.94)	1.01 (0.95-1.08)
7-12	0.59 (0.57-0.62)	0.63 (0.61-0.65)	0.68 (0.66-0.70)	0.66 (0.62-0.69)	0.76 (0.70-0.82)
≥13	0.55 (0.52-0.57)	0.56 (0.54-0.57)	0.62 (0.60-0.64)	0.60 (0.57-0.64)	0.70 (0.63-0.77)
**Number of inpatient visits[b]**					
0	Reference	Reference	Reference	Reference	Reference
1-3	1.03 (0.99-1.07)	1.04 (1.02-1.07)	1.02 (1.00-1.05)	0.98 (0.94-1.03)	1.01 (0.92-1.12)
≥3	0.95 (0.86-1.05)	0.91 (0.83-0.99)	0.94 (0.87-1.01)	0.99 (0.87-1.14)	0.83 (0.55-1.25)
**Number of antihypertensive medications**					
1	Reference	Reference	Reference	Reference	Reference
2	0.98 (0.95-1.01)	0.99 (0.97-1.00)	1.04 (1.02-1.06)	1.02 (0.99-1.05)	0.98 (0.93-1.04)
≥3	1.11 (1.07-1.14)	1.09 (1.07-1.11)	1.19 (1.16-1.21)	1.12 (1.08-1.16)	1.11 (1.04-1.18)

NHB = Non-Hispanic Black, NHW = Non-Hispanic White, API = Asian/Pacific Islander, Other = Native American/Alaskan Native and multiple or other races and ethnicities

[a]Adjusted for all variables in the table

[b]Visits in 2018

© 2023 Harrison TN et al. *JAMA Network Open.*

eFigure 3. **Age-Adjusted Proportion of Patients with Blood Pressure Control (<130/80 mm Hg) by Race/Ethnicity Among Adults in the Kaiser Permanente Southern California Hypertension Registry, 2008-2019**

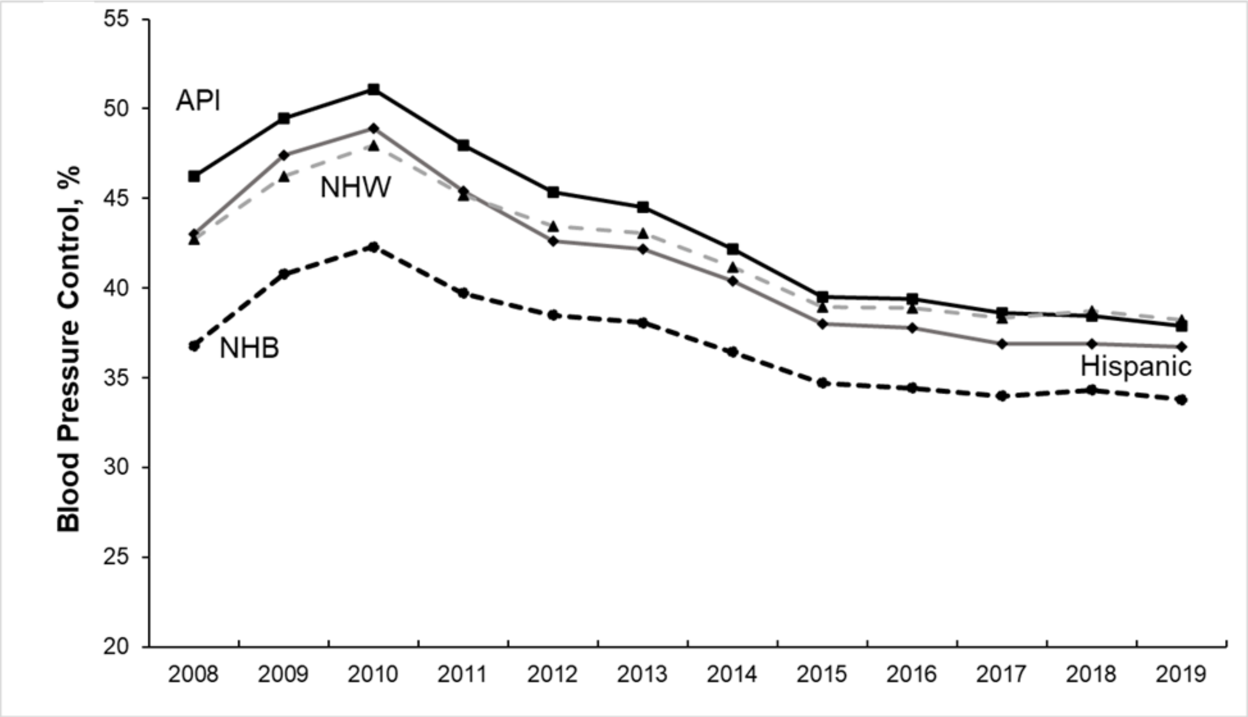

NHB = Non-Hispanic Black, NHW = Non-Hispanic White, API = Asian/Pacific Islander

© 2023 Harrison TN et al. *JAMA Network Open.*
