# Supplementary material for: Blood Pressure Control Among Black and White Adults Following a Quality Improvement Program in a Large Integrated Health System
Source: JAMA Netw Open. 2023 Jan 6;6(1):e2249930. doi: 10.1001/jamanetworkopen.2022.49930 (PMC9856959; doi:10.1001/jamanetworkopen.2022.49930)
Supplement: Supplement 2. — Data Sharing Statement [file jamanetwopen-e2249930-s002.pdf]

Data Sharing Statement

Harrison. Blood Pressure Control Among Black and White Adults Following a Quality Improvement Program in a Large Integrated Health System. *JAMA Netw Open*. Published January 06, 2023. doi:10.1001/jamanetworkopen.2022.49930

Data
Data available: No
